# A Dual‐Phase Exosomal Nanotherapy Enhances Continual Efferocytosis by Coordinating Checkpoint Inhibition and Metabolic Reprogramming in Atherosclerosis

**DOI:** 10.1002/advs.75941

**Published:** 2026-06-04

**Authors:** Danwen Zheng, Shiteng Cai, Jinfeng Gao, Sheng Zhao, Bohan Wei, Xueyi Weng, Zhengmin Wang, Qiaozi Wang, Qiyu Li, Chengzhi Han, Weiyan Li, Yiwen Tan, Yuyuan Fu, Meng Ji, Zheyong Huang, Yanan Song, Juying Qian, Junbo Ge

**Affiliations:** ^1^ Department of Cardiology Shanghai Institute of Cardiovascular Diseases Zhongshan Hospital Fudan University Shanghai China; ^2^ State Key Laboratory of Cardiovascular Diseases Zhongshan Hospital Fudan University Shanghai China; ^3^ NHC Key Laboratory of Ischemic Heart Diseases Shanghai China; ^4^ Institute of Biomedical Sciences Fudan University Shanghai China

**Keywords:** Arg1, efferocytosis, exosomes, metabolic reprogramming, SIRPa

## Abstract

Atherosclerosis is a chronic inflammatory disease characterized by defective efferocytosis, which contributes to necrotic core expansion and plaque instability. This dysfunction arises from two key barriers: first, impaired recognition of apoptotic cells due to activation of the CD47–SIRPα immune checkpoint; and second, insufficient metabolic processing of apoptotic cell‐derived substrates, which limits the capacity for continual efferocytosis. Arg1‐mediated arginine metabolism has emerged as a crucial pathway supporting this process. To address these limitations, we created Am@SExo, a dual‐functional engineered exosome derived from macrophages that unites checkpoint inhibition with metabolic reprogramming. The vesicles overexpress SIRPα on its surface to competitively engage CD47 on apoptotic cells and relieve the inhibitory signal, facilitating initial binding and uptake. Subsequently, it delivers Arg1 mRNA to recipient macrophages, driving an arginine to putrescine program aligned with Rac1 and actin remodeling to sustain successive rounds of clearance. In ApoE^−/−^ mice, systemic administration of Am@SExo significantly reduced necrotic core area, increased fibrous cap thickness, and enhanced features of plaque stability. Together, our findings demonstrate that Am@SExo as a single‐platform, dual‐phase modulator that restores macrophage continual efferocytosis, offering a promising strategy to resolve inflammation and stabilize atherosclerotic plaques.

## Graphical abstract

Here we developed Am@SExo, a dual‐functional engineered exosome that coordinates CD47–SIRPα checkpoint blockade with Arg1 mRNA–mediated metabolic reprogramming. Surface SIRPα promotes recognition of apoptotic cells and plaque targeting, while Arg1 expression enhances arginine–ornithine metabolism, Rac1 activation, and actin remodeling in macrophages. This strategy restores continual efferocytosis and improves atherosclerotic plaque resolution and stability.

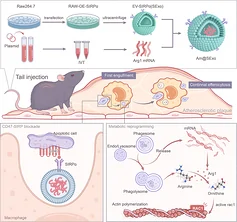

## 1 Introduction

Atherosclerosis is a chronic inflammatory disease of arterial walls [1], contributing to severe clinical events such as myocardial infarction, stroke, and peripheral artery disease [2, 3, 4]. Although lipid‐lowering therapies have significantly reduced cardiovascular events, a substantial residual risk remains [5], largely driven by persistent vascular inflammation that is not adequately addressed by lipid‐lowering agents. Recent anti‐inflammatory strategies [6, 7] further support the need for inflammation‐targeted interventions beyond lipid control.

Growing evidence places efferocytosis at the center of inflammatory control in atherosclerotic plaques. Under physiological conditions, macrophages clear apoptotic cells in repeated cycles, which promotes resolution and preserves tissue homeostasis. In advanced lesions, this program fails, apoptotic and necrotic debris accumulate, the necrotic core expands, the fibrous cap weakens, and the risk of rupture and thrombosis increases [8]. Restoring continual efferocytosis is a promising approach to stabilize vulnerable plaques and reduce residual cardiovascular risk.

A key recognition‐stage brake on efferocytosis is the CD47–SIRPα checkpoint. In atherosclerotic plaques, abnormally high CD47 strengthens SIRPα signaling and impairs macrophage recognition and uptake [8, 9]. Anti‐CD47 antibodies can enhance efferocytosis in preclinical studies, yet systemic use is constrained by hematologic toxicities due to the broad expression of CD47 on healthy cells [10]. These considerations motivate local or selective modulation of the CD47–SIRPα axis to relieve the checkpoint while minimizing systemic burden.

However, while checkpoint relief improves recognition and uptake, maintaining continual efferocytosis is often limited after engulfment by the need to metabolize substantial apoptotic‐cell cargo [11], including amino acids [12]. A central node is arginine allocation between inducible nitric oxide synthase (iNOS) and arginase‐1 (Arg1) [13]. Processing through iNOS sustains inflammatory signaling. In contrast, Arg1 converts apoptotic‐cell‐derived arginine into ornithine and polyamines, which are compatible with Rac1‐dependent actin remodeling and successive rounds of engulfment [14]. In atherosclerotic plaques, lesional macrophages often show reduced Arg1, shifting arginine use toward iNOS and further compromising continual efferocytosis [15]. Taken together, these observations motivate dual, complementary interventions that both relieve the CD47–SIRPα checkpoint at recognition and reinforce Arg1‐linked metabolic support after engulfment to sustain continual efferocytosis.

Macrophage‐derived exosomes provide a biocompatible vehicle with innate tropism for inflamed tissues and can display proteins and deliver mRNA cargo [16, 17, 18, 19]. Here, we developed a dual‐functional engineered exosome named Am@SExo that integrates immune checkpoint inhibition with metabolic reprogramming to restore defective efferocytosis. Surface SIRPα competitively engages CD47 to neutralize the “don't‐eat‐me” checkpoint, conferring selective binding to atherosclerotic plaques and promoting their uptake by macrophages. Simultaneously, the exosomes deliver Arg1 mRNA to recipient macrophages, driving an arginine to putrescine metabolic program and supports continual efferocytosis. Am@SExo preferentially accumulates in atherosclerotic plaques, enhances continual efferocytosis, reduces plaque burden and necrotic core, and improves features of plaque stability, highlighting its promise as a nanotherapeutic for atherosclerosis.

## 2 Results

### 2.1 Arg1 Insufficiency is Associated With Reduced Continual Efferocytosis in Atherosclerotic Macrophages

**FIGURE 1:**
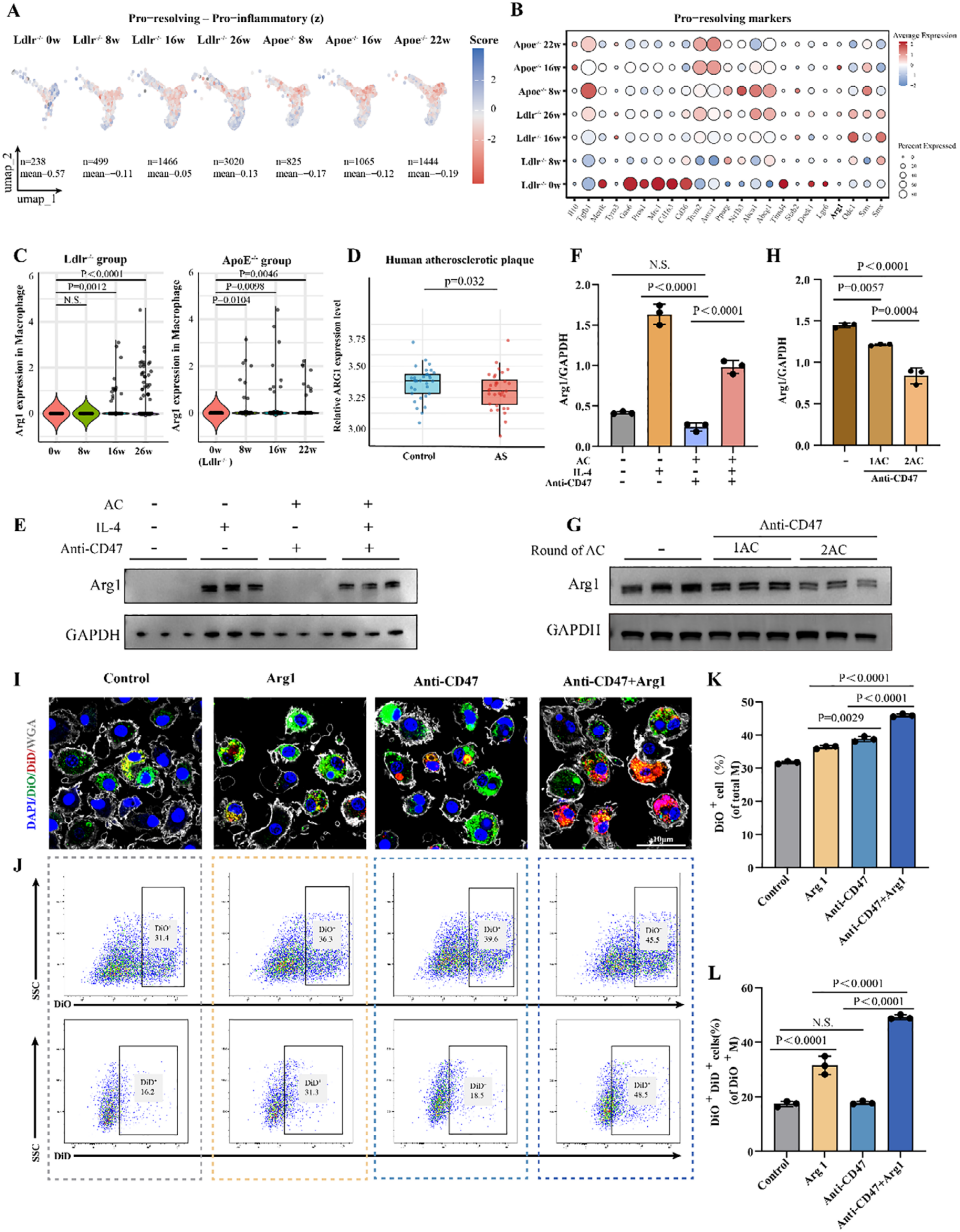
Arg1 insufficiency is associated with reduced continual efferocytosis in atherosclerotic macrophages. (A) Net pro‐resolving module score of macrophages during atherosclerosis progression. (B) The expression levels of representative pro‐resoving markers across progression. (C) Expression levels of Arg1 in macrophages at different time points during atherosclerosis progression, based on the GSE155513 database. (D) Different expression of ARG1 between atherosclerotic plaque tissues and adjacent normal tissues in the GSE43292 database. (E) Western blot analysis of Arg1 protein levels in BMDMs treated with PBS, IL‐4, CD47 antibody + apoptotic cells (AC), or IL‐4+CD47 antibody + AC. (F) Quantification of Arg1 protein levels from (E). Statistical analysis was calculated using one‐way ANOVA and Tukey's tests (n = 3). (G) Western blot analysis of Arg1 protein expression in BMDMs pretreated with IL‐4 and subsequently treated with CD47‐antibody, followed by one or two rounds of efferocytosis (1AC, 2AC). (H) Quantification of Arg1 protein levels from (G). Statistical analysis was calculated using one‐way ANOVA and Tukey's tests (n = 3). (I) Representative CLSM images, with corresponding flow cytometry (J) and quantification (K, L), of BMDMs undergoing two rounds of efferocytosis under the indicated conditions (Control, Arg1, Anti‐CD47, and Anti‐CD47+Arg1). First‐round apoptotic cells were labeled with DiO (green) and second‐round cells with DiD (red). Scale bar, 10 µm. Statistical analysis was calculated using the one‐way ANOVA and Tukey's tests (n = 3).

We analyzed the macrophage state dynamics using publicly available datasets (GSE155513) (Figure 1A). During the progression of atherosclerosis, we observed a gradual decline in pro‐resolving macrophage scores, which are associated with tissue repair and efferocytosis. This trend was consistent in both Ldlr^−/−^ and ApoE^−/−^ mouse models. Analysis of pro‐resolving genes showed a broad attenuation of resolution‐associated programs as atherosclerosis progressed (Figure 1B). Among these markers, Arg1 remained relatively low expression compared with other pro‐resolving genes and showed only modest fluctuations over time, with a slight downward shift in the later stages of disease (Figure 1C). These patterns suggest that Arg1 constitutes a comparatively weak and only minimally inducible component of the lesional resolution program, which may become further constrained as lesions advance. In human plaques (GSE43292), ARG1 was significantly downregulated in diseased versus adjacent normal tissue (Figure 1D), indicating cross‐species concordance. Given the known role of Arg1 in generating polyamines that support cytoskeletal remodeling during efferocytosis [14, 20], these observations suggest that Arg1 insufficiency may reduce the conversion of arginine to polyamine and thereby dampen continual efferocytosis.

To further evaluate the role of Arg1 in efferocytosis, we first examined whether efferocytosis could directly induce Arg1 expression in vitro. CD47 blockade is a commonly used method to promote efferocytosis [10]. Western blot showed that simply using a CD47 antibody to increase engulfment did not significantly elevate Arg1 (Figure 1E). By contrast, IL‐4 robustly induced Arg1, consistent with its role as a canonical pro‐resolving stimulus. Notably, however, when IL‐4–primed macrophages were subjected to successive rounds of apoptotic cell uptake, Arg1 expression progressively declined with each additional efferocytic cycle (Figure 1G). This pattern indicates that Arg1 becomes further reduced as efferocytic load increases, suggesting that the metabolic capacity supporting continual efferocytosis may be progressively strained under sustained demand, particularly within the inflammatory and metabolically stressed microenvironment of atherosclerotic lesions.

Next, we investigated whether Arg1 expression levels could influence continual efferocytosis. We transfected BMDMs with Arg1 mRNA and performed continual efferocytosis assays. Confocal microscopy and flow cytometry analyses showed that Arg1 overexpression alone significantly enhanced continual efferocytic efficiency, rising from 17.37% ± 1.01% to 31.50% ± 3.35% (Figure 1I–L). Our previous work demonstrated that blocking the CD47–SIRPα axis enhances efferocytosis in a single‐round phagocytosis model [21, 22]. In this study, we identified that CD47 blockade alone did not improve continual efferocytosis (17.83% ± 0.59%). Combining Arg1 with CD47 blockade produced a cooperative effect, elevating continual efferocytosis to 49.13% ± 0.84% (Figure 1K,L).

Together, these data indicate that Arg1 boosts continual efferocytosis on its own and confers additional benefit in the presence of CD47 blockade, supporting the effectiveness of the combined strategy.

### 2.2 Synthesis and Characterization of EVs Loaded With mRNA

**FIGURE 2:**
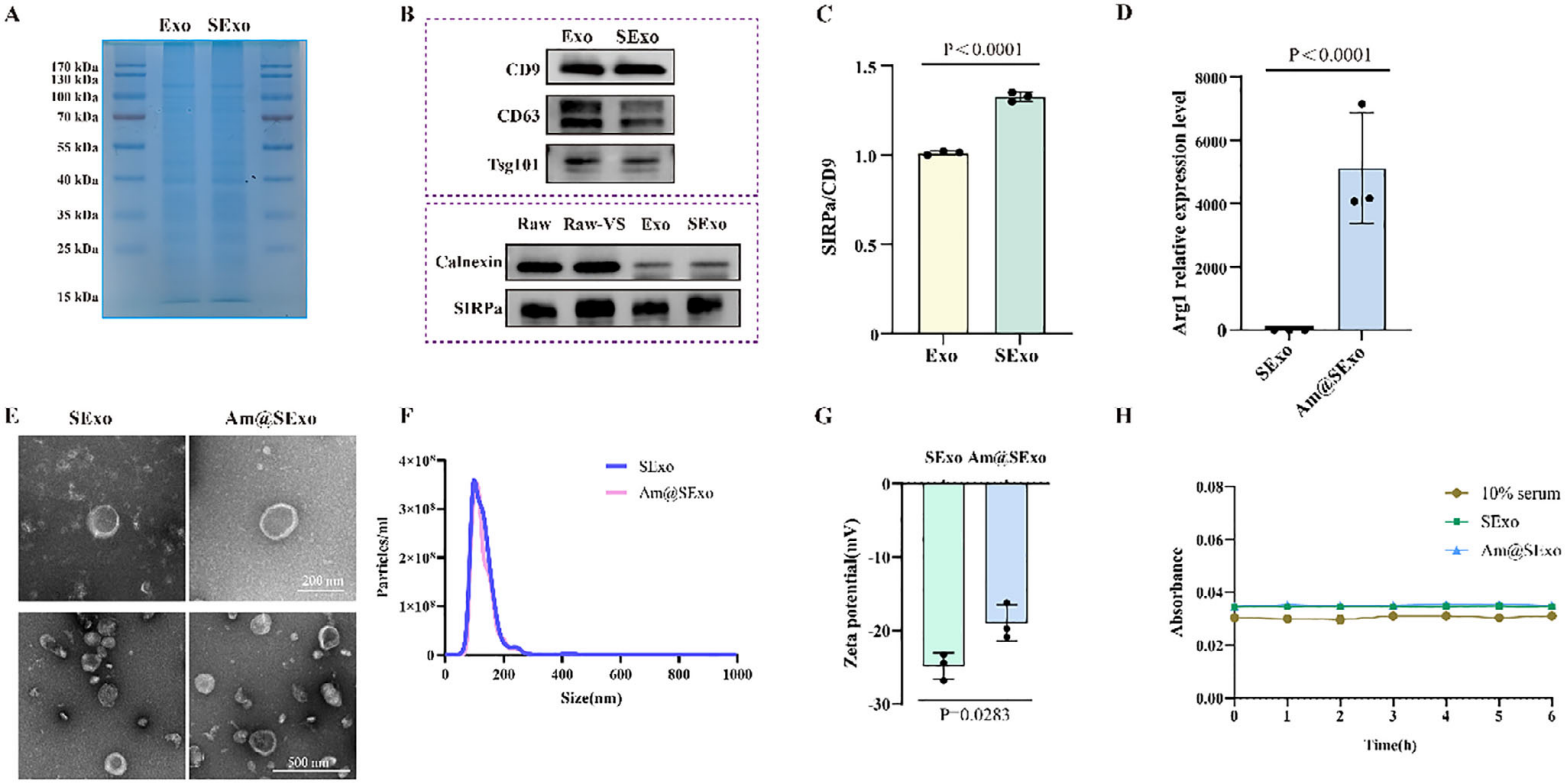
Synthesis and characterization of EVs loaded with mRNA. (A) Coomassie blue staining of Exo and SExo. (B) Western blot analysis showing the expression of EV markers (CD9, CD63, Tsg101), along with Calnexin and SIRPα. (C) Western blot quantification of SIRPα overexpression in SExo. Statistical analysis was calculated using the two‐sided Student's t‐test (n = 3). (D) qPCR analysis of Arg1 mRNA encapsulation in Am@SExo. Statistical analysis was calculated using the two‐sided Student's t‐test (n = 3). (E) Representative TEM images of SExo and Am@SExo. Scale bars, 200 nm (upper panels) and 500 nm (lower panels). (F) Size distribution of SExo and Am@SExo measured by NTA (n = 3). (G) Zeta potentials of SExo, and Am@SExo measured by DLS. Statistical analysis was calculated using the two‐sided Student's t‐test (n = 3). (H) Absorbance of 10% human plasma alone, SExo, and Am@SExo in 10% human plasma at 37°C over time, measured at 590 nm (n = 3).

As illustrated in the Graphical Abstract, SIRPα‐overexpressing Raw264.7 cells (Raw‐OE‐SIRPa) were generated via lentiviral transduction. Western blotting (Figure S1) and immunofluorescence staining (Figure S2) confirmed the successful SIRPα overexpression. Extracellular vesicles (EVs) were then isolated from control and SIRPα‐overexpressing cells, yielding Exo and SExo, respectively. Coomassie blue staining (Figure 2A) revealed comparable protein profiles between Exo and SExo. Western blot analysis (Figure 2B) confirmed the presence of classical EV markers CD9, CD63, and Tsg101 in both groups. Minimal Calnexin contamination was observed, indicating good purity. Additionally, SIRPα expression was retained in SExo, as demonstrated by Western blotting (Figure 2C).

Arg1 mRNA (Am) was synthesized via in vitro transcription (IVT) (Figure S3) and subsequently loaded into Exo and SExo using a commercial EV transfection kit, generating Am@Exo and Am@SExo, respectively. The average encapsulation efficiencies of Am@Exo and Am@SExo (Figure S4) were 82.33% ± 0.70% and 81.67% ± 0.73%, respectively, with no statistically significant difference between groups. Quantitative PCR analysis (Figure 2D) further confirmed the successful encapsulation of Arg1 mRNA in Am@SExo.

Transmission electron microscopy (TEM) (Figure 2E) and nanoparticle tracking analysis (NTA) (Figure 2F) showed that both SExo and Am@SExo exhibited typical spherical morphology with uniform particle size distributions, averaging 104.7 ± 7.0 and 107.3 ± 4.9 nm, respectively. These results indicate that mRNA loading had minimal impact on the morphology and size distribution of SExo. Dynamic light scattering (DLS) analysis (Figure 2G) revealed that, compared with SExo, the zeta potential of Am@SExo shifted from −24.86 ± 1.8 to −18.95 ± 2.44 mV, became less negative. This observation is consistent with the partial neutralization of anionic membrane sites by cationic constituents present in the Exo Fect formulation, along with the localization of mRNA–carrier complexes in close proximity to the vesicle surface, thereby altering the zeta potential [23].

The stability and protective effect of EV‐encapsulated mRNA were further evaluated. Incubation in 10% serum showed that the particle size of Am@SExo remained stable over 24 h, and its PDI was comparable to that of SExo (Figure S5A,B). Agarose gel electrophoresis further showed that naked Arg1 mRNA was rapidly degraded in 10% serum or RNase A, whereas EV‐encapsulated mRNA remained detectable after prolonged incubation, indicating an effective protective effect of the vesicles on the mRNA cargo (Figure S5C). To evaluate colloidal stability under physiological conditions, SExo and Am@SExo were incubated in 10% human plasma and monitored by absorbance over time (Figure 2H). The relatively stable absorbance values, together with the blank plasma control, suggested that Am@SExo did not show obvious plasma‐induced aggregation under the tested conditions, further supporting their stability in biological environments. Taken together, the net‐negative surface charge after loading and the absence of plasma‐induced aggregation support the colloidal stability of Am@SExo under the tested conditions.

Collectively, these results confirm the successful construction of Am@SExo with high mRNA loading efficiency, preserved EV morphology and size, and favorable surface charge and serum stability properties.

### 2.3 Targeted Delivery of Engineered EVs

**FIGURE 3:**
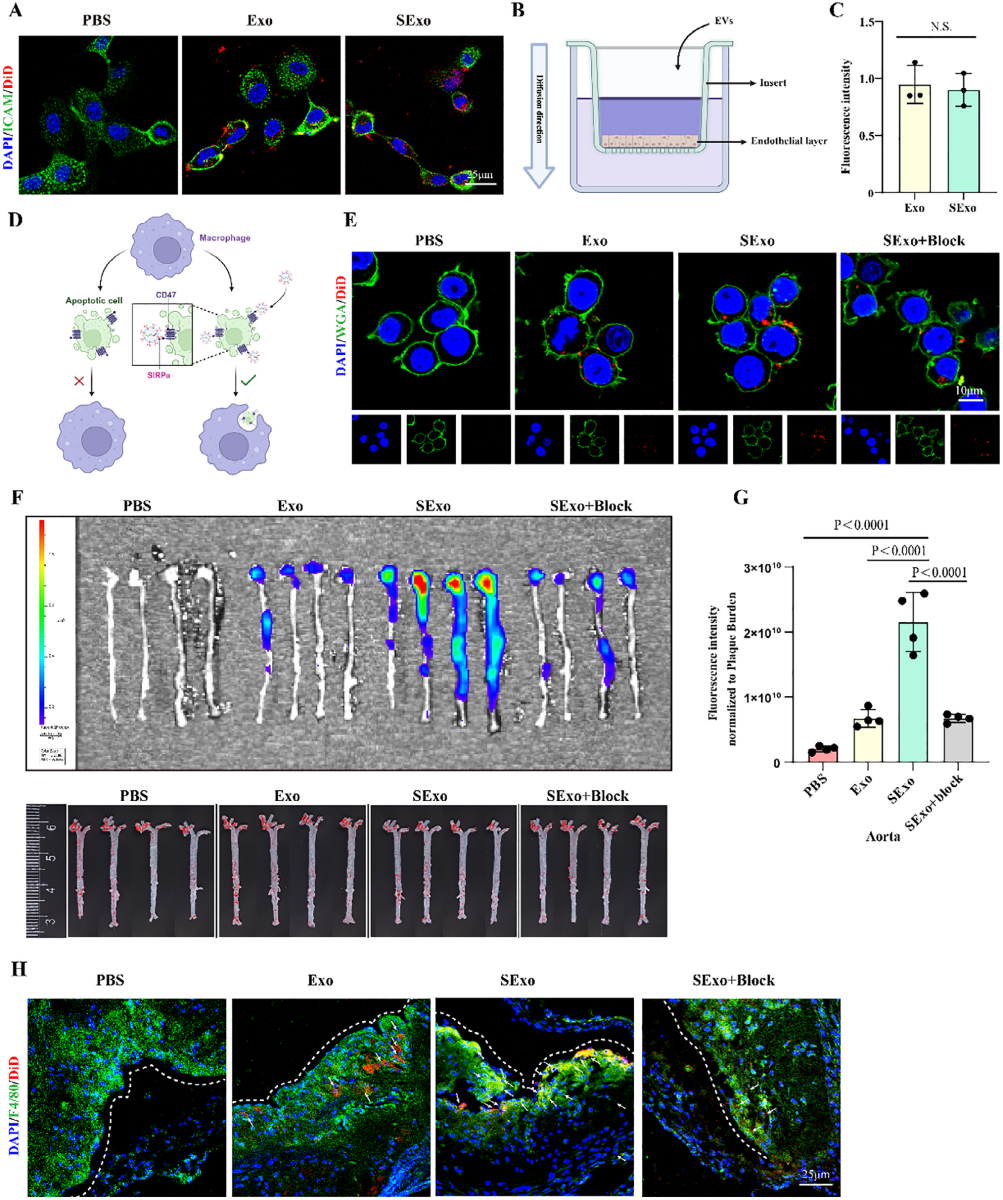
Targeted delivery of Engineered EVs. (A) Representative CLSM images showing the binding of DiD‐labeled Exo and SExo to LPS‐pretreated HUVECs. Scale bar, 25 µm. (B) Schematic of the Transwell migration assay. (C) Quantification of fluorescence intensity of migrated EVs in the lower chamber. Statistical analysis was calculated using the two‐sided Student's t‐test (n = 3). (D) Schematic illustration of the design rationale for SIRPα‐engineered EVs to enhance the recognition and efferocytosis of apoptotic cells by blocking the CD47–SIRPα axis. (E) Representative immunostaining images of apoptotic cells (ACs) incubated with PBS or DiD‐labeled EVs for 15 min. Scale bar, 10 µm. (F) Ex vivo IVIS images of aortas harvested 2 h after intravenous injection of PBS or DiD‐labeled EVs into 12‐week Western diet (WD)‐fed ApoE^−/−^ mice. (G) Quantification of fluorescence intensity in aortas, normalized to plaque burden. Statistical analysis was calculated using the one‐way ANOVA and Tukey's multiple comparison tests. (n = 4). (H) Immunofluorescence analysis of the colocalization of DiD‐labeled EVs (red) and F4/80^+^ macrophages (green) in the aortic root. Nuclei were stained with DAPI (blue). White arrows indicate the colocalization of EVs with F4/80. Scale bar, 25 µm.

To evaluate the targeting ability of engineered EVs, we first examined their adhesion to inflammation‐associated cells in vitro. Human umbilical vein endothelial cells (HUVECs) were pre‐treated with lipopolysaccharide (LPS) for 24 h to induce an inflammatory phenotype, followed by incubation with DiD‐labeled Exo or SExo. Confocal microscopy qualitatively showed efficient membrane adhesion of both EV types to HUVECs, with comparable fluorescence signals between groups (Figure 3A). Similar results were observed in Raw264.7 cells (Figure S6A) and bone marrow‐derived macrophages (BMDMs) (Figure S6B), indicating comparable targeting capacities. In line with this, both EV types retained key parent cell‐derived adhesion and chemotaxis‐related proteins, including CD18, CCR2, and CXCR2 (Figure S7). To assess the transendothelial transport capacity of EVs, we established a Transwell‐based migration model using 0.4 µm pore inserts (Figure 3B). Confluent HUVEC monolayers were stimulated with LPS, and DiD‐labeled EVs were added to the upper chamber. Fluorescence intensity (Figure 3C) in the lower chamber was measured to quantify EVs translocation. No significant difference was observed between Exo and SExo, indicating comparable transendothelial transport efficiency in vitro. Together, these results indicate that SIRPα engineering did not markedly alter the baseline endothelial interaction or transendothelial transport properties of macrophage‐derived EVs.

We then examined whether SIRPα engineering conferred selective binding to ACs, which are abundant in atherosclerotic plaques. Figure 3D schematically illustrates the proposed interaction between SIRPα‐engineered EVs and CD47 on ACs. After 30 min of incubation with DiD‐labeled EVs, confocal imaging showed that SExo exhibited markedly greater accumulation on AC membranes than Exo, and this advantage was largely abolished by SIRPα‐blocking treatment (Figure 3E), supporting a CD47–SIRPα‐mediated interaction.

We next investigated whether this property translated into enhanced plaque targeting in vivo. DiD‐labeled Exo, SExo, and SExo pre‐treated with a SIRPα‐blocking antibody (SExo + Block) were intravenously injected into ApoE^−/−^ mice fed a Western diet for 12 weeks. At 2 h post‐injection, the aorta (Figure 3F) and major organs (heart, liver, spleen, lung, kidney, and brain) (Figure S8A) were harvested for ex vivo fluorescence imaging. SExo exhibited the highest relative fluorescence accumulation in the aorta (Figure 3G), with a 3.21 and 3.20‐fold increase compared to the Exo and SExo + Block groups, respectively. To account for hepatic clearance, we calculated the aorta‐to‐liver fluorescence ratio (Figure S8C), which was significantly higher in the SExo group, further confirming its enhanced plaque‐targeting capability. Aortic‐root staining further confirmed higher accumulation of SExo within atherosclerotic lesions, with clear colocalization with F4/80^+^ macrophages (Figure 3H). In addition, Cy5‐labeled mRNA‐loaded EVs showed that, compared with Am@Exo, Am@SExo delivered more cargo into the lesion area and exhibited clearer colocalization with lesional macrophages (Figure S8D), supporting effective in vivo delivery to the plaque macrophage‐rich microenvironment.

To further assess potential off‐target interactions with viable circulating CD47^+^ cells, we analyzed the distribution of DiD‐labeled vesicles among peripheral blood cells at 2 h after intravenous injection (Figure S9). Flow‐cytometric analysis showed that the highest DiD^+^ fraction was observed in CD11b^+^ myeloid cells, reaching 11.10% ± 0.65% in the Am@Exo group and 11.33% ± 0.35% in the Am@SExo group. In contrast, the erythrocyte‐associated signal remained much lower, at 2.58% ± 0.34% and 2.81% ± 0.17%, respectively, with no significant difference between the two groups. These results indicate that although a fraction of vesicles can associate with circulating blood cells shortly after systemic administration, Am@SExo did not show disproportionate erythrocyte association under our experimental conditions. Meanwhile, the limited CD11b^+^ signal suggests that early interaction with circulating myeloid cells may occur, but does not appear to indicate marked depletion of Am@SExo in the bloodstream before plaque accumulation.

Overall, Exo and SExo showed similar adhesion and transendothelial translocation. Higher surface SIRPa on SExo conferred stronger binding and enrichment at CD47‐high apoptotic cells and plaques, further improving targeting of atherosclerotic lesions.

### 2.4 In Vitro Functional Assessment and Enhancement of Continual Efferocytosis

**FIGURE 4:**
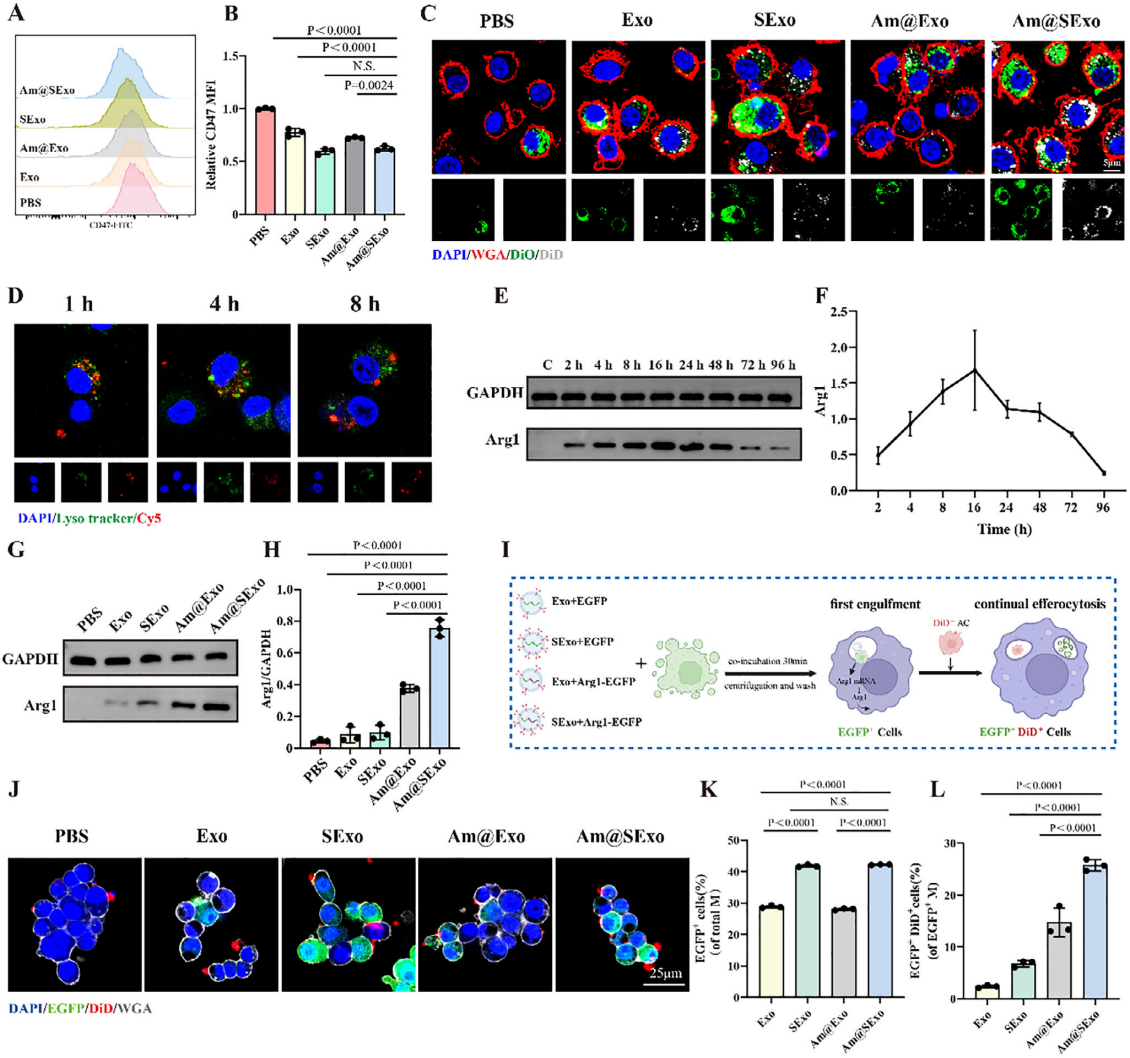
In vitro functional assessment and enhancement of continual efferocytosis. (A) Flow cytometry histograms showing CD47 fluorescence intensity of apoptotic cells after incubation with different EVs. (B) Quantification of CD47 MFI, Statistical analysis was calculated using the one‐way ANOVA and Tukey's tests (n = 3). (C) Representative CLSM images showing colocalization of DiO‐labeled ACs and DiD‐labeled EVs in bone marrow–derived macrophages (BMDMs). Scale bar, 5 µm. (D) CLSM images showing intracellular trafficking of Cy5‐labeled mRNA (red) in BMDMs at 1, 4, and 8 h post‐incubation. Lysosomes were labeled with LysoTracker (green), nuclei with DAPI (blue). Scale bar, 5 µm. (E) Time‐change Western blot of Arg1 protein expression in BMDMs after Am@SExo treatment. (F) Line graph showing the time‐dependent expression of Arg1 protein (n = 3). (G) Western blot comparing Arg1 expression at 16 h across different EV treatment groups. (H) Quantification of Arg1/GAPDH ratio at 16 h, Statistical analysis was calculated using the one‐way ANOVA and Tukey's tests (n = 3). (I) Schematic illustration of the in vitro efferocytosis assay. EGFP^+^ cells were considered to have undergone single efferocytosis, while EGFP^+^DiD^+^ cells indicated continual efferocytosis. (J) Representative CLSM images of Raw264.7 cells undergoing two rounds of efferocytosis. Scale bar, 25 µm. (K, L) Flow cytometry quantification of single (EGFP^+^) and continual (EGFP^+^DiD^+^) efferocytosis in Raw264.7 cells. Statistical analysis was calculated using the one‐way ANOVA and Tukey's tests (n = 3).

To assess the functional properties of engineered EVs, we first evaluated their ability to block CD47 on apoptotic cells (ACs). ACs were co‐incubated with PBS, Exo, SExo, Am@Exo, or Am@SExo for 30 min. Unbound EVs were removed by centrifugation to obtain AC–EV complexes (AC–EVs), and surface CD47 mean fluorescence intensity (MFI) on ACs was measured by flow cytometry. As shown in the histograms (Figure 4A) and quantification (Figure 4B), both SExo and Am@SExo significantly reduced CD47 MFI relative to PBS, indicating effective blockade of CD47. Notably, loading Arg1 mRNA did not impair the CD47‐blocking capacity of SExo. These findings indicate that EV surface overexpression of SIRPα enhances competitive binding to CD47 on ACs, thereby disrupting the CD47–SIRPα “don't‐eat‐me” signal and facilitating phagocytosis. Next, to determine whether macrophages could efficiently internalize AC–EV complexes, we incubated the complexes with BMDMs for 2 h. Confocal images revealed increased intracellular co‐localization of ACs and EVs in the SExo and Am@SExo groups (Figure 4C), supporting enhanced uptake by macrophages following CD47 blockade. Flow cytometry further confirmed that SExo and Am@SExo were taken up by BMDMs more efficiently than Exo and Am@Exo, respectively, with no significant difference between SExo and Am@SExo (Figure S10).

To assess endosomal escape of delivered mRNA, Cy5‐labeled Arg1 mRNA was loaded into EVs and incubated with BMDMs. CLSM imaging (Figure 4D) showed that at 1 h the Cy5 signal largely overlapped with LysoTracker‐positive lysosomes, indicating endosomal entrapment. By 4 h, partial separation of Cy5 from lysosomes was evident. By 8 h, a substantial fraction of Cy5 was lysosome‐independent, indicating effective endosomal escape and cytoplasmic release.

To evaluate mRNA translation, AC–EV complexes were again used to deliver mRNA into BMDMs. Western blot analysis (Figure 4E) revealed a gradual increase in Arg1 protein levels following Am@SExo treatment, peaking at 16 h and remaining elevated up to 72 h before declining at 96 h. Densitometric quantification (Figure 4F) confirmed this temporal expression pattern. Furthermore, at the 16‐h peak, both Am@Exo and Am@SExo significantly upregulated Arg1 expression compared to PBS, Exo, and SExo (Figure 4G,H), with Am@SExo inducing the highest level, indicating superior mRNA delivery efficiency.

To evaluate continual efferocytosis, we used the scheme in Figure 4I. Exo and SExo were loaded with EGFP mRNA, while Am@Exo and Am@SExo carried Arg1–EGFP mRNA. AC–EV complexes were prepared as described and incubated with Raw264.7 cells or BMDMs for 16 h to allow the first round of efferocytosis. Subsequently, the macrophages were exposed to DiD‐labeled apoptotic cells for an additional 2 h to assess a second round of uptake. The continual efferocytosis efficiency was defined as the proportion of EGFP^+^DiD^+^ macrophages among EGFP^+^ macrophages. We first performed the two‑round efferocytosis assay in Raw264.7 cells. Confocal imaging (Figure 4J) qualitatively indicated that Am@SExo exhibited the highest continual efferocytosis efficiency. Flow cytometry (Figure 4K,L) further quantified these observations. For continual efferocytosis, Am@SExo achieved continual efferocytosis efficiencies that were 10.93‐, 3.82‐, and 1.75‐fold higher than those of the Exo, SExo, and Am@Exo groups, respectively, highlighting its superior ability to sustain efferocytosis over multiple rounds.

Taken together, Am@SExo acts as a dual‑stage enhancer of efferocytosis. Surface SIRPα disrupts the CD47 checkpoint to facilitate initial uptake, while Arg1 mRNA enhances the macrophage's capacity for continual clearance. Similar results were observed in BMDMs (Figures S11 and S12), confirming the generalizability of these findings across different macrophage models.

### 2.5 In Vitro Reprogramming of the Macrophage Inflammatory Microenvironment and Metabolic State

**FIGURE 5:**
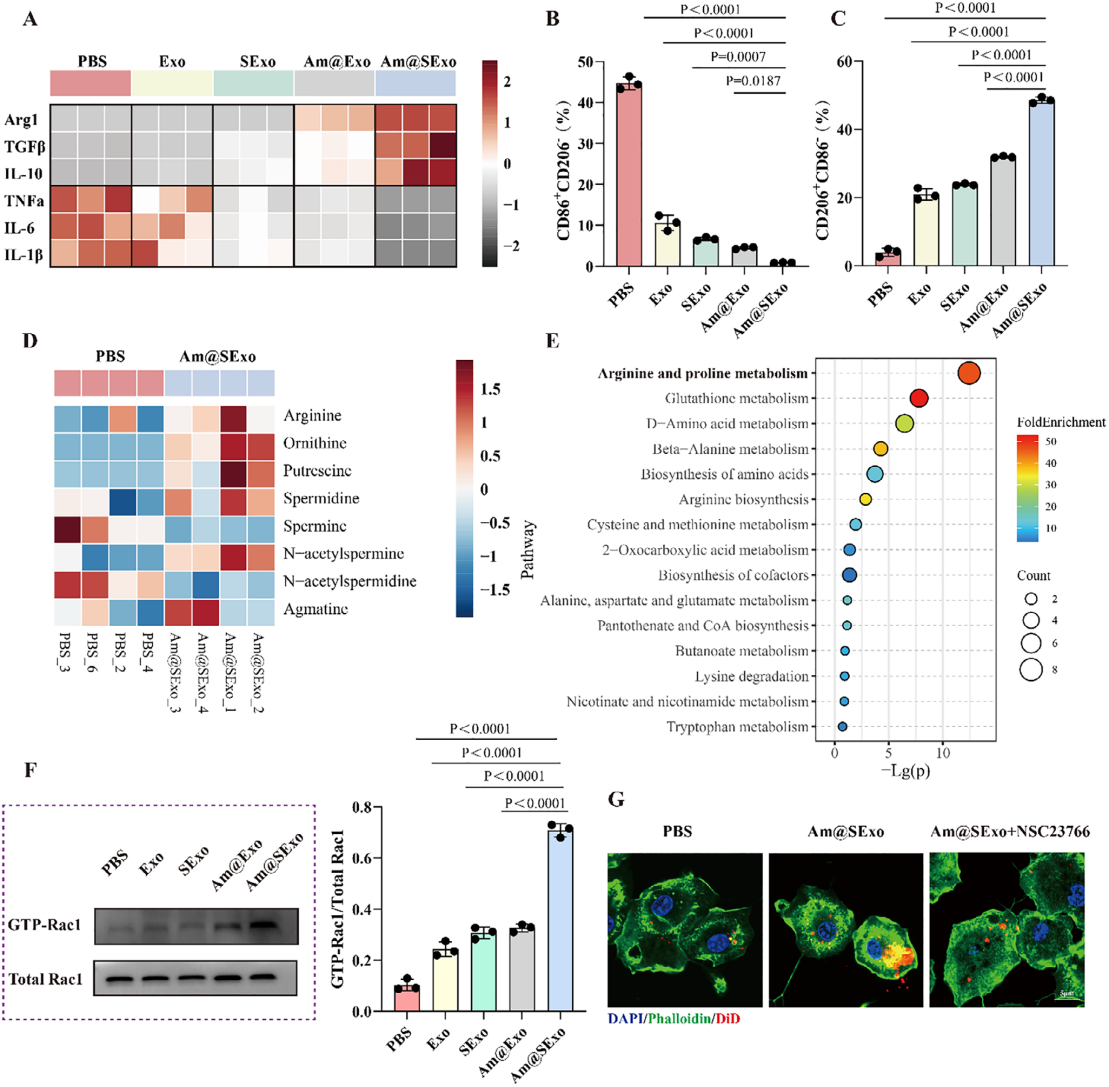
In vitro reprogramming of macrophage inflammatory microenvironment and metabolic state. (A) qPCR heatmap of cytokines in BMDMs after efferocytosis under the indicated treatments (PBS, Exo, SExo, Am@Exo, Am@SExo). Pro‐inflammatory genes: TNF‐α, IL‐1β, IL‐6. Anti‐inflammatory genes: TGF‐β, IL‐10. Metabolic marker: Arg1. Statistical analysis was calculated using the one‐way ANOVA and Tukey's tests (n = 3). (B, C) Flow cytometry analysis of BMDM polarization after efferocytosis. Statistical analysis was calculated using the one‐way ANOVA and Tukey's tests (n = 3). (D) Targeted metabolomics heatmap of arginine/polyamine‐pathway metabolites. Statistical analysis was calculated using the one‐way ANOVA and Tukey's tests (n = 4). (E) KEGG pathway enrichment analysis of differentially regulated metabolites. (F) Rac1 activation assay in BMDMs by PAK‐PBD pull‐down of GTP‐Rac1. Total Rac1 was detected from input lysates. Statistical analysis was calculated using the one‐way ANOVA and Tukey's tests (n = 3). (G) Representative CLSM images of actin‐mediated engulfment of DiD‐labeled apoptotic cells (ACs) in BMDMs treated with PBS, Am@SExo, or Am@SExo + NSC23766. Scale bar, 5 µm.

Given that lesional macrophages in atherosclerosis are predominantly pro‐inflammatory [24], promoting their transition toward a pro‐resolving phenotype is a promising therapeutic strategy. We first assessed the expression of inflammatory cytokines by qPCR (Figure 5A, Figure S13). Arg1 mRNA levels were significantly increased in Am@Exo and Am@SExo groups, confirming successful mRNA delivery. Importantly, Am@SExo treatment upregulated pro‐resolving cytokines (TGF‐β, IL‐10) and downregulated pro‐inflammatory cytokines (TNF‐α, IL‐1β, IL‐6), highlighting its potent immunomodulatory effect. We next assessed polarization. Flow cytometry analysis (Figure 5B,C; Figure S14) revealed that BMDMs co‐incubated with ACs alone exhibited high CD86 expression, indicative of a pro‐inflammatory state. Treatment with Exo, SExo, Am@Exo, or Am@SExo all promoted macrophage polarization toward a CD206^+^ pro‐resolving phenotype, with Am@SExo showing the most pronounced effect.

We next examined metabolism. Targeted metabolomics showed that Am@SExo increased ornithine and putrescine (Figure 5D; Figure S15), and KEGG enrichment highlighted arginine and proline metabolism (Figure 5E), consistent with activation of the Arg1–ornithine decarboxylase (ODC)–putrescine pathway as anticipated [14]. In addition, the metabolite pattern featured higher spermidine and lower spermine, together with increased N‐acetylspermine and decreased N‐acetylspermidine (Figure 5D; Figure S15). This pattern is in line with prior reports of efferocytosis‐linked polyamine flux and turnover [25, 26]. Consistent with the metabolomic data, qPCR showed increased ODC and Rac1 transcripts in the Am@SExo group (Figure S16).

Under the same conditions, PAK‐PBD pull‐down detected the highest Rac1‐GTP levels with Am@SExo (Figure 5F). The Rac1 inhibitor NSC23766 [27] reduced uptake of DiD‐labeled apoptotic cells after Am@SExo treatment (Figure 5G), supporting a role for Rac1‐dependent actin remodeling in the observed enhancement of continual efferocytosis.

In conclusion, Am@SExo enhances continual efferocytosis in vitro, by an Arg1–ODC–putrescine–Rac1/actin axis.

### 2.6 In Vivo Anti‐Atherosclerotic Effect

**FIGURE 6:**
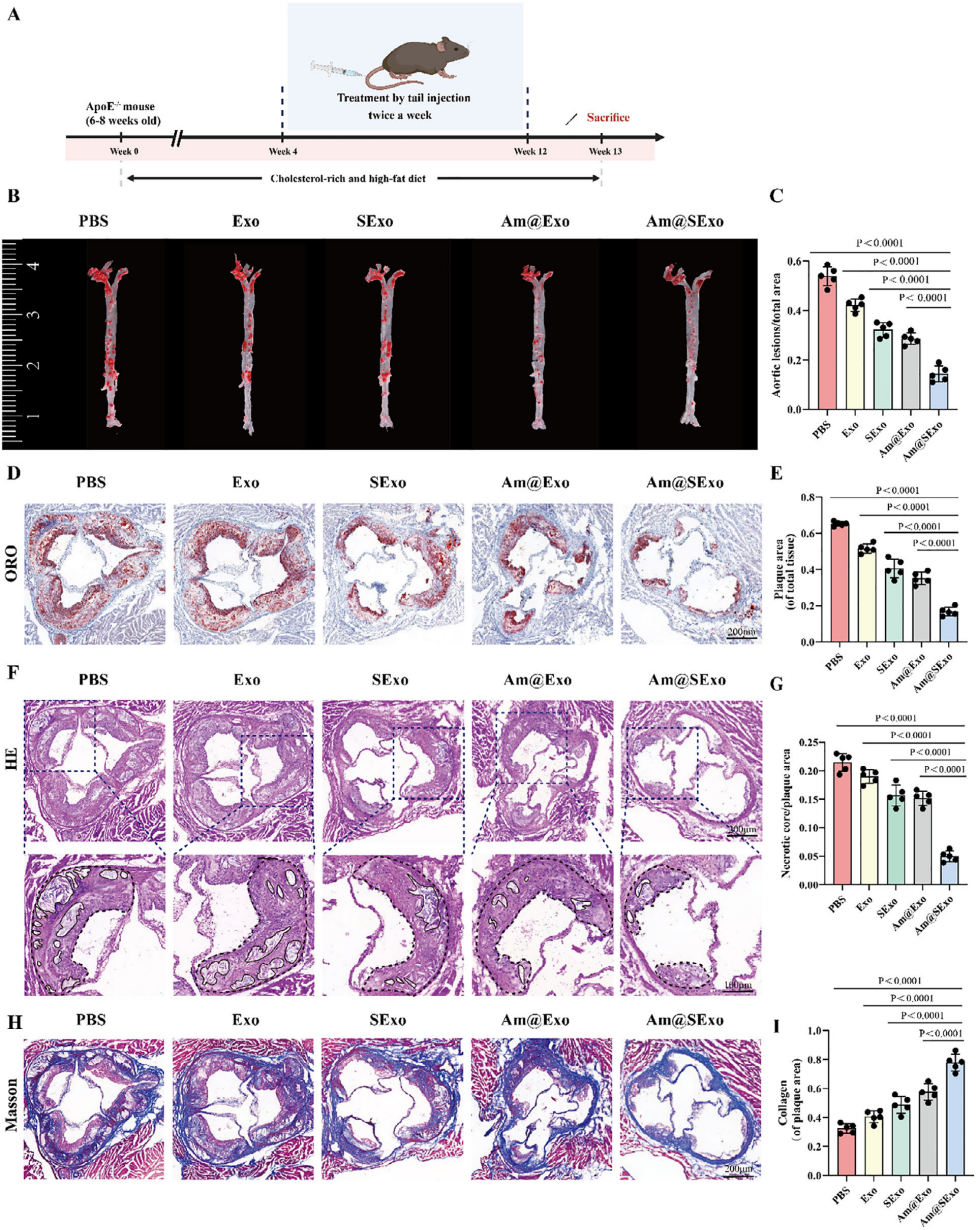
In vivo anti‐atherosclerotic effects of Am@SExo in ApoE^−/−^ mice. (A) Schematic illustration of the experimental protocol. From week 4, ApoE^−/−^ mice received intravenous injections of PBS, Exo, SExo, Am@Exo, or Am@SExo twice weekly for 8 weeks. At week 13, mice were sacrificed for tissue analysis. (B, C) Representative Oil Red O (ORO) staining images of aortas and quantification of plaque area (% of total area). Statistical analysis was calculated using the one‐way ANOVA and Tukey's tests (n = 5). (D, E) ORO staining of aortic root cross‐sections and quantification of plaque area (% of total tissue area). Scale bar, 200 µm. Statistical analysis was calculated using the one‐way ANOVA and Tukey's tests (n = 5). (F, G) Representative Hematoxylin and eosin (H&E) staining of aortic root sections, plaque areas, and necrotic cores are outlined by black dashed lines and black solid lines, respectively, with quantification of the necrotic core‐to‐plaque area ratio. Scale bar, 200 and 100 µm. Statistical analysis was calculated using the one‐way ANOVA and Tukey's tests (n = 5). (H, I) Representative Masson's trichrome staining of aortic root sections showing collagen deposition (blue), with quantification of collagen content in plaques. Scale bar, 200 µm. Statistical analysis was calculated using the one‐way ANOVA and Tukey's tests (n = 5).

Previous studies have demonstrated that elevated Arg1 expression contributes to plaque regression and fibrous cap stabilization in atherosclerosis [28]. To evaluate the therapeutic efficacy of Am@SExo in vivo, we established an atherosclerotic mouse model (Figure 6A). ApoE^−/^‐ mice were fed a Western‐type high‐fat/high‐cholesterol diet for 12 weeks. Starting at week 5, mice received tail vein injections twice weekly for 8 weeks. At week 13, the mice were sacrificed, and the aorta and aortic root were collected for histological analysis.

Oil Red O (ORO) staining of aortas (Figure 6B,C; Figure S17A) showed that Am@SExo treatment markedly reduced plaque area (14.37% ± 2.52%) compared to the PBS (53.90% ± 2.73%), Exo (42.15% ± 1.76%), SExo (32.21% ± 2.46%), and Am@Exo (28.62% ± 1.63%) groups. Similar trends were observed in the aortic root sections (Figure 6D,E; Figure S17B), where plaque areas accounted for 65.19% ± 0.93%, 51.57% ± 1.81%, 40.54 ± 4.01%, 35.11% ± 2.67%, and 16.74% ± 1.76% of the total tissue area in the PBS, Exo, SExo, Am@Exo, and Am@SExo groups, respectively.

Hematoxylin and eosin (H&E) staining (Figure 6F,G) further revealed that Am@SExo significantly reduced the necrotic core‐to‐plaque area ratio, indicating decreased plaque vulnerability. Quantitatively, the necrotic core fraction was 21.46% ± 1.31%, 18.99% ± 0.95%, 15.65% ± 1.31%, 15.16% ± 0.97%, and 4.97% ± 0.70% in the PBS, Exo, SExo, Am@Exo, and Am@SExo groups, respectively, with Am@SExo showing the lowest value. In addition, Masson's trichrome staining (Figure 6H,I) showed a marked increase in collagen content within plaques of the Am@SExo‐treated group, suggesting enhanced plaque stability.

Collectively, these findings demonstrate that Am@SExo exerts potent anti‐atherosclerotic effects in vivo by reducing plaque burden, stabilizing plaque structure, and promoting inflammation resolution.

### 2.7 Study of the Antiatherosclerotic Mechanism

**FIGURE 7:**
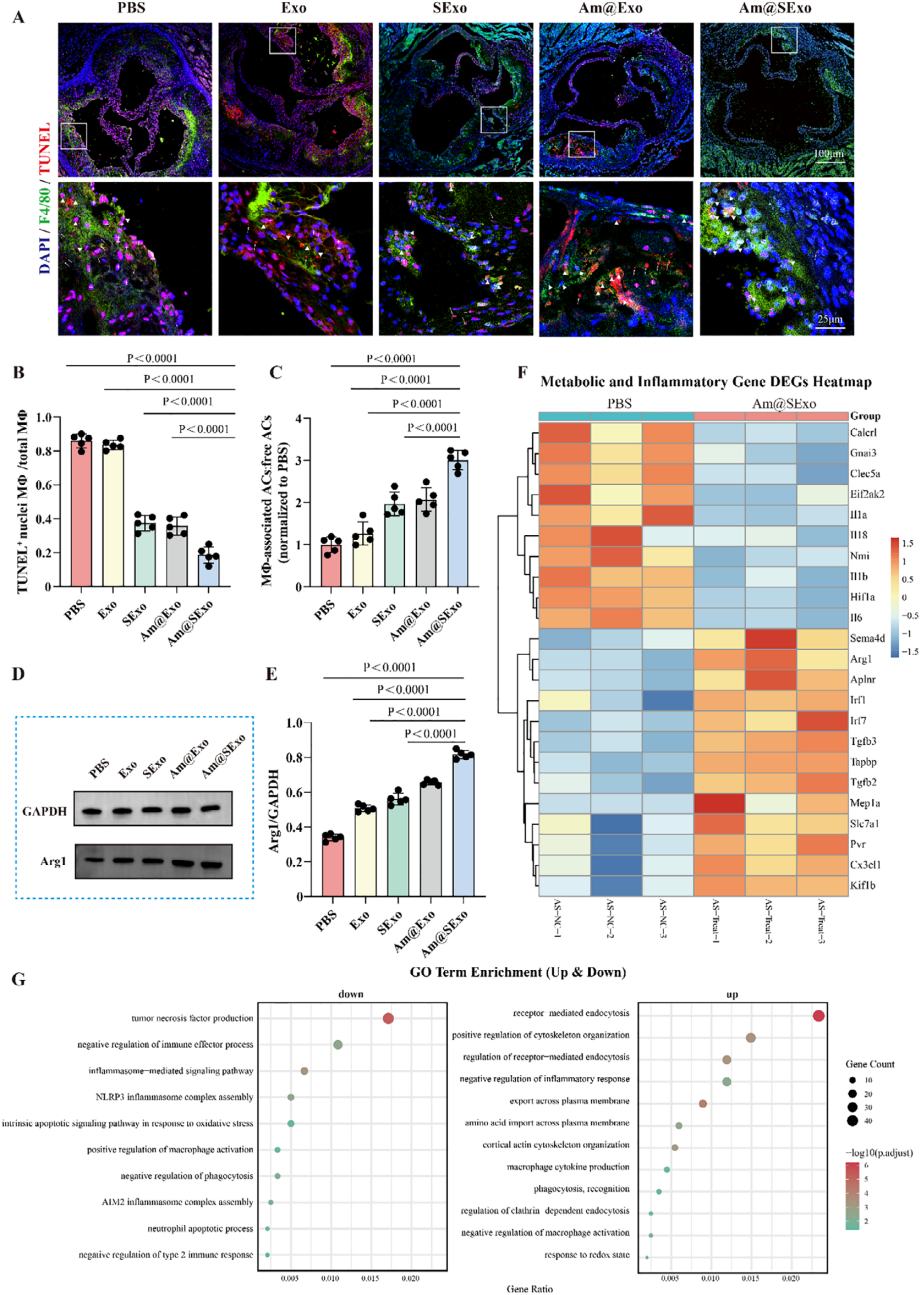
Am@SExo enhances macrophage efferocytosis and reduces inflammation in atherosclerotic plaques. (A) Representative immunofluorescence images of aortic root sections stained with TUNEL (red), F4/80 (green), and DAPI (blue). TUNEL‐positive nuclei located inside F4/80^+^ macrophages are counted as apoptotic macrophages (TUNEL^+^ nuclei MΦ; “+”), whereas TUNEL‐positive nuclei adjacent to or surrounded by F4/80^+^ macrophages are counted as macrophage‐associated ACs (“△”), and those without any contact with F4/80^+^ macrophages are counted as free ACs (“→”). Scale bars, 100 µm (top), 25 µm (bottom). (B) Quantification of apoptotic macrophages (TUNEL^+^ nuclei MΦ) per section. Statistical analysis was calculated using the one‐way ANOVA and Tukey's tests (n = 5). (C) Ratio of macrophage‐associated ACs to free ACs, representing efferocytosis efficiency. Statistical analysis was calculated using the one‐way ANOVA and Tukey's tests (n = 5). (D) Western blot analysis of Arginase‐1 (Arg1) expression in aortic tissues collected 3 days after the final injection. (E) Quantification of Arg1 protein levels normalized to GAPDH. Statistical analysis was calculated using the one‐way ANOVA and Tukey's tests (n = 5). (F) Heatmap of differentially expressed inflammation‐ and metabolism‐related genes identified by bulk RNA sequencing of aortic tissues from PBS‐ and Am@SExo‐treated mice (n = 3). (G) Gene Ontology (GO) enrichment analysis of upregulated and downregulated pathways.

TUNEL staining (Figure 7A) was performed to assess macrophage apoptosis and efferocytic activity within atherosclerotic plaques. Definitions of the TUNEL categories are provided in the figure legend. The PBS group showed a high proportion of apoptotic macrophages and a low ratio of macrophage‐associated to free apoptotic cells, consistent with impaired efferocytosis. In contrast, SExo, Am@Exo, and especially Am@SExo reduced the proportion of apoptotic macrophages and increased the macrophage‐associated/free AC ratio (Figure 7B,C), indicating improved efferocytic capacity. To further explore the underlying mechanisms, we examined the expression of Arg1, in aortic tissues. Western blot (Figure 7D,E) revealed that Arg1 expression was significantly upregulated in the Am@SExo group compared to the PBS group, supporting the induction of a pro‐resolving macrophage phenotype.

To gain broader insight into the anti‐atherosclerotic mechanisms of Am@SExo, we performed bulk RNA sequencing on aortic tissues from PBS‐ and Am@SExo‐treated mice. Differentially expressed genes (DEGs) (Figure 7F) associated with inflammation showed that Am@SExo treatment led to the downregulation of multiple pro‐inflammatory genes and the upregulation of pro‐resolving genes, including Arg1. Further Gene Ontology Biological Process (GO BP) enrichment analysis (Figure 7G) identified significant suppression of inflammatory pathways such as TNF signaling, while pathways related to endocytosis, actin cytoskeleton organization, and negative regulation of inflammatory response were significantly enriched among the upregulated genes. These pathways are closely associated with enhanced efferocytosis and inflammation resolution.

Taken together, these results demonstrate that Am@SExo functions as a dual‐action enhancer, which, through SIRPα overexpression and Arg1 mRNA delivery, orchestrates multiple stages of macrophage efferocytosis. This leads to reduced macrophage apoptosis, enhanced clearance of apoptotic cells, and promotion of an pro‐resolving microenvironment, ultimately contributing to plaque stabilization in atherosclerosis.

### 2.8 In Vivo Safety Evaluation

Finally, we assessed the systemic safety of Am@SExo following 8 weeks of treatment in Apo E^−/−^ mice. Three days after the final tail vein injection, mice from both the PBS and Am@SExo groups were euthanized, and major organs including the heart, liver, spleen, lungs, kidneys, and brain were collected for analysis. As shown in Figure S18, H&E staining of major organs revealed no signs of abnormal inflammation or histopathological damage. These results indicate that Am@SExo exhibits good biocompatibility and does not induce systemic toxicity in mice, supporting its safety for in vivo applications.

## 3 Discussion

Defective continual efferocytosis remains a key barrier to plaque resolution. Continual efferocytosis requires coordinated recognition, uptake, and metabolic and cytoskeletal readiness for subsequent rounds. In advanced plaques this program is impaired at two linked points. Apoptotic cells aberrantly express CD47 that engages macrophage SIRPα and restricts recognition and uptake. After engulfment macrophages face a substantial metabolic load and Arg1 insufficient limits conversion of arginine to ornithine and polyamines which are needed for Rac1 dependent actin remodeling and successive rounds of clearance [8, 14]. These constraints lead to accumulation of apoptotic cargo and amplification of inflammation.

We therefore advance an integrated intervention that restores recognition and the metabolic and cytoskeletal preparedness required for successive rounds of clearance. We developed Am@SExo, a macrophage derived dual‐functional engineered exosome that addresses both recognition and metabolic readiness. Surface overexpression of SIRPα enables competitive engagement of CD47 on apoptotic cells and facilitates initial uptake. Arg1, a key enzyme that converts apoptotic cell‐derived arginine to ornithine, is consistently low in lesional macrophages and becomes further suppressed as disease progresses. Prior studies have linked Arg1 deficiency to impaired continual efferocytosis [14]. Evidence presented here indicates that repeated apoptotic‐cell uptake did not increase Arg1, whereas supplementation with Arg1 enhanced continual efferocytosis. After macrophages internalize CD47‐positive apoptotic cells bound by Am@SExo, Arg1 mRNA delivery reprograms macrophage arginine metabolism after engulfment and supports Rac1‐dependent actin remodeling for multiple rounds of clearance. In atherosclerotic mice this dual action is associated with smaller plaque burden, reduced necrotic core, and features of greater stability.

Importantly, the therapeutic effect of Am@SExo depends not only on its dual‐functional cargo design, but also on a stepwise targeting and delivery process within the plaque microenvironment. As a macrophage‐derived exosome platform, Am@SExo retains inflammation‐associated endothelial interaction and the ability to cross the vascular barrier, which may facilitate lesion access. Within plaques, surface‐overexpressed SIRPα enables preferential binding of the engineered vesicles to CD47‐positive apoptotic cells, thereby enriching the vesicles at sites of defective efferocytosis. Once bound to apoptotic cells, the vesicle‐associated cargo can then enter lesional macrophages together with apoptotic‐cell engulfment during efferocytosis. In this way, the platform is designed to couple plaque access, apoptotic‐cell targeting, and macrophage‐directed cargo delivery into a coherent therapeutic route. After efferocytosis‐associated uptake, endo/lysosomal escape becomes a critical downstream step for functional mRNA delivery by Am@SExo. In the present study, the time‐dependent reduction in colocalization between Cy5‐labeled mRNA and LysoTracker‐positive compartments, together with detectable Arg1 protein expression in recipient macrophages, supports that a fraction of the delivered mRNA was able to escape the endo/lysosomal pathway and remain functionally available for translation. Based on previous studies, several factors may contribute to this process. First, efferocytosis is not merely a degradative event, but a highly regulated intracellular trafficking program [29]. Second, phagosomes and endo/lysosomal compartments undergo dynamic maturation and fusion events, which may, under certain conditions, provide opportunities for partial cargo escape during membrane remodeling [30]. Third, extracellular vesicles possess intrinsic membrane‐protective and membrane‐interaction properties, which may help preserve cargo integrity and facilitate intracellular membrane crossing under certain conditions [31]. The precise molecular mechanism responsible for this process, however, remains to be further investigated in future studies using more specialized approaches, such as live‐cell imaging, endosomal rupture reporters, subcellular fractionation, and perturbation of candidate EV membrane proteins or lipid components.

This stepwise targeting and intracellular delivery process also highlights an important distinction between Am@SExo and most existing single‐target strategies aimed at improving efferocytosis. Most existing strategies to enhance efferocytosis are single‐target, such as anti‐CD47 antibodies [10] for recognition or LNP‐IL‐10 [32] for macrophage phenotype. In contrast, Am@SExo was designed to act within the plaque efferocytosis axis itself, simultaneously improving recognition of apoptotic cells and restoring the metabolic and cytoskeletal readiness required for subsequent rounds of clearance. By combining CD47–SIRPα checkpoint interference with Arg1 mRNA‐driven metabolic reprogramming, this platform may provide broader therapeutic effects. In addition, compared with synthetic nanocarriers, the endogenous exosome‐based carrier may offer favorable biocompatibility and disease‐relevant interactions with inflamed lesions, which could further support its therapeutic applicability in atherosclerosis [33]. Although inclusion of direct comparator groups such as anti‐CD47 antibody treatment would have further strengthened head‐to‐head evaluation, such expansion would have required substantial additional animal use and resources. Therefore, in the present proof‐of‐concept study, we focused on establishing the mechanistic and therapeutic rationale of the dual‐functional exosome platform itself.

From a translational perspective, the present proof‐of‐concept system still faces practical challenges for further development. The relatively modest SIRPα overexpression achieved in donor macrophages suggests that donor‐cell engineering remains to be optimized. Future studies may further improve SIRPα enrichment by optimizing donor‐cell engineering strategies, such as electroporation‐assisted nucleic acid delivery, improved transduction conditions, or other active exosome‐loading approaches. In parallel, large‐scale clinical translation will require solutions for the limited yield and batch heterogeneity of conventional 2D culture systems, together with GMP‐compatible purification and robust release criteria for identity, purity, potency, sterility, and stability [34]. Future development will therefore require both improved donor‐cell engineering and advances in scalable EV manufacturing, such as closed bioreactor‐based culture systems, serum‐free or chemically defined media, and standardized downstream purification workflows [35] in line with evolving EV standardization frameworks such as MISEV2023 [36]. These advances will be important for the eventual development of clinical‐grade engineered exosome therapeutics.

Moreover, combining this strategy with standard lipid lowering therapy or low‐dose anti‐inflammatory treatments may further reduce residual risk linked to defective efferocytosis, which warrants systematic evaluation in future studies.

## 4 Conclusion

This study reveals that Arg1 expression remains persistently low in atherosclerotic plaques and does not increase with multi‐round efferocytosis, while supplementing Arg1 restores macrophage continual efferocytosis. Building on this, we developed Am@SExo, an engineered exosome that combines CD47 SIRPα immune checkpoint blockade with arginine metabolic reprogramming. This strategy effectively corrects continual efferocytosis defects, mitigates inflammation, promotes in vivo plaque regression and stability, and demonstrates promising translational potential for atherosclerosis therapy.

## 5 Experimental Section

### 5.1 Animals and Cell Lines

Apo E^−/−^ mice (6–8 weeks old) were purchased from Shanghai Jiesijie Laboratory Animal Co. Animal experiments were approved by the Ethics Committee of Zhongshan Hospital, Shanghai, China (2025‐015). At the study endpoint, mice were anesthetized with isoflurane, and tissues were harvested and stored for downstream analyses. BMDMs were isolated as described previously [37]. Briefly, after mice were euthanized by rapid cervical dislocation, their femurs and tibias were quickly separated, and then the bone marrow cavity was flushed with sterile PBS through a 70 µm cell strainer to obtain a single cell suspension. The isolated bone marrow cells were centrifuged and resuspended in DMEM containing 10% FBS, 1% penicillin/streptomycin (P/S), and 20 ng/mL recombination murine macrophage colony stimulating factor (M‐CSF, PeproTech, USA) for 7 days to obtain mature macrophages. Raw264.7, Jurkat, and HUVEC cell lines were purchased from the Institute of Biochemistry and Cell Biology, Chinese Academy of Sciences (Shanghai China).

### 5.2 SIRPa Overexpression

Lentiviral vectors are widely used for stable and efficient gene delivery in hard‐to‐transfect cell [38]. SIRPa overexpression (pLV‐EF1a‐ Sirpa‐CMV‐EGFP‐T2A‐Puro) and vector lentivirus (pLV‐EF1a‐ MCS‐CMV‐EGFP‐T2A‐Puro), were purchased from Lifespan (Shanghai, China). The Sirpa coding sequence in the overexpression vector was synthesized based on the validated mouse RefSeq transcript NM_001355158.2, which encodes SIRPα isoform 1 (NP_001342087.1). According to the current NCBI annotation, multiple transcript variants (including variants 1, 8, 11, and 12) encode the same isoform 1 protein. Raw264.7 were transduced with these lentivirus particles according to the manufacturer's instructions. Western blot analysis and immunofluorescence staining confirmed successful overexpression of SIRPα.

### 5.3 Isolation and Quantification of Exo and SExo

Raw264.7 cells and SIRPα‐overexpressing Raw264.7 cells were cultured in 10 cm plates until reaching appropriate confluency, followed by incubation in serum‐free medium for 24 h. Extracellular vesicles were isolated from the culture supernatants of Raw264.7 and SIRPα‐overexpressing cells, and designated as Exo and SExo, respectively. The conditioned medium was then collected and subjected to sequential centrifugation at 4°C(300 × g for 10 min, 2000 × *g* for 10 min, and 10 000 × g for 30 min)to remove cells and debris. The supernatant was subsequently filtered through a 0.22 µm filter. The filtrate was concentrated using a 100 kDa molecular weight cut‐off centrifugal filter and further subjected to ultracentrifugation at 100 000 × *g* for 60 min at 4°C using a Beckman Coulter Optima XPN‐80 ultracentrifuge (Miami, FL, USA). The resulting exosome pellet was resuspended in pre‐chilled phosphate‐buffered saline (PBS) and stored at −80°C for subsequent experiments. The morphology of the exosomes was characterized using transmission electron microscopy (TEM). Protein concentrations in the samples were quantified using a BCA protein assay kit (Thermo Scientific). Nanoparticle tracking analysis (NTA) [NanoSight NS300 (Malvern Panalytical) and ZetaSizer Pro (Malvern Panalytical)] was used to measure particle concentration, size distribution, and zeta potential of the exosome samples. The isolated Exo and SExo were estimated to contain 4.09 × 10^9^ and 2.38 × 10^9^ particles per 10 mL of supernatant, respectively, or 50.18 µg and 28.14 µg of protein. Exosome identity and SIRPα overexpression were confirmed by western blot analysis.

### 5.4 Preparation of Modified mRNA

Arg1, EGFP, and Arg1‐EGFP fragments were assembled into the expression vector using the 2x MultiF Seamless Assembly Mix (ABclonal). The assembled products were transformed into Escherichia coli competent cells. Transformed bacteria were plated onto LB agar plates containing ampicillin and incubated to allow colony formation. Single colonies were subsequently inoculated into LB liquid medium (Miller formulation) and cultured overnight with vigorous shaking at 250 rpm. The correct insertion and sequence of the plasmids were confirmed by Sanger sequencing. Plasmids were extracted using the Endo‐free Plasmid Mini Kit II (omega), and DNA concentration was measured using a NanoDrop 2000 spectrophotometer (Thermo). The DNA template was obtained using a rapid endonuclease(reBio). DNA templates were purified using a DNA Extraction Reagent (ACMEC) and analyzed by agarose gel electrophoresis. In vitro transcription of mRNA [39] was performed using T7 RNA polymerase (Hzymes) according to the manufacturer's instructions. The synthesized mRNA was purified using lithium chloride precipitation. After quantification with a NanoDrop 2000 spectrophotometer (Thermo), all mRNA samples were diluted to the desired concentration with nuclease‐free water and stored at −80°C until use.

### 5.5 Preparation and Characterization of Am@Exo and Am@SExo

The assembly of mRNA with extracellular vesicles (EVs) was performed using the Exo‐Fect Exosome Transfection Kit (System Biosciences) according to the manufacturer's instructions. Briefly, 10 µL of Exo‐Fect solution, 20 µL of nucleic acid solution (containing 1 µg of mRNA), and 70 µL of purified exosomes were mixed in a 1.5 mL microcentrifuge tube. The mixture was incubated at 37°C in a shaker for 10 min, followed by immediate placement on ice to halt the reaction. Subsequently, 30 µL of ExoQuick‐TC reagent was added to terminate the transfection process. The mixture was centrifuged at 13 000–14 000 rpm for 3 min in a microcentrifuge. The resulting pellet, containing the mRNA‐loaded exosomes, was resuspended in phosphate‐buffered saline (PBS). The concentration of unencapsulated mRNA remaining in the supernatant was measured to calculate the encapsulation efficiency. The successful loading of Arg1 mRNA into EVs was validated by quantitative PCR (qPCR). For subsequent in vitro and in vivo experiments, mRNA‐loaded EVs were normalized and administered based on EV protein content as measured by BCA assay.

### 5.6 Am@SExo Stability Assessment

Naked and EV‐encapsulated mRNA were exposed to FBS and RNase A, and their stability was evaluated using agarose gel electrophoresis. We incubated 1 µg of naked Arg1 mRNA or Am@SExo with 10% (v/v) FBS or RNase A (10 ng/mL) at 37°C for the specified durations. After incubation, the samples were subjected to electrophoresis on a 2% agarose gel containing GelRed. The gel was imaged using the ChemiDoc imaging system (Bio‐Rad). To assess serum stability, SExo and Am@SExo were resuspended in 10% human plasma and incubated at 37°C for 6 h. The absorbance at 590 nm was measured every hour using an Epoch 2 microplate spectrophotometer (BioTek, USA), with blank 10% human plasma included as a control. In addition, the particle size of Am@SExo was monitored over time in 10% serum, and the PDI of SExo and Am@SExo was measured under the indicated conditions.

### 5.7 The Binding Ability of EVs to the Raw264.7, BMDM, and HUVEC In Vitro

Raw264.7 cells, bone marrow‐derived macrophages (BMDMs), and human umbilical vein endothelial cells (HUVECs) were seeded at a density of 1.5 × 10^5^ cells per well in confocal culture dishes. After 24 h of culture in their respective complete media, HUVECs were pretreated with lipopolysaccharide (LPS, 0.5 µg/mL) for 24 h to induce an inflammatory phenotype. Cells were washed with PBS and incubated with 200 µL of DiD‐labeled Exo or SExo (50 µg EV/10^6^ cells) for 30 minutes at 37°C. Following incubation, cells were fixed with 4% paraformaldehyde for 20 minutes at room temperature and washed thoroughly with PBS. To block nonspecific binding, cells were incubated with 5% BSA in PBS for 1 h. The dishes were incubated with primary antibodies overnight at 4°C. Then, the dishes were washed and incubated with secondary antibodies. Detailed information on the antibodies used is provided in Table S3. The samples were imaged using an Olympus FV3000 confocal laser scanning microscope. To evaluate the targeting efficiency of Exo and SExo to HUVECs in vitro, we use a 0.4 µm transwell plate (Millipore, USA) to mimic the environment. HUVECs were seeded in the upper chamber. After pretreatment with LPS (0.5 µg/mL) for 24 h, DiD‐labeled Exo or SExo was added to the upper chamber at the same dose of 50 µg EV protein per 10^6^ cells. After that, the medium in the lower chambers were collected, and the flurescence intensity was measured using a microplate reader.

### 5.8 Biodistribution and Plaque‐Targeting Assessment

For EV biodistribution analysis, mice were intravenously injected with PBS, DiD‐labeled Exo, DiD‐labeled SExo, or DiD‐labeled SExo with blocking treatment at an EV dose of 200 µg protein per mouse. At 2 h after injection, the aorta and major organs, including the heart, liver, spleen, lung, kidney, and brain, were harvested for ex vivo fluorescence imaging using an IVIS imaging system (PerkinElmer, Inc., Waltham, MA). Aortic root sections were further collected to evaluate plaque targeting. Sections were stained with DAPI and F4/80, and the colocalization of DiD‐labeled EVs with plaque macrophages was analyzed by confocal microscopy. For mRNA delivery assessment, mice were intravenously injected with PBS, Cy5‐mRNA‐loaded Exo (Am@Exo), or Cy5‐mRNA‐loaded SExo (Am@SExo) at a dose of 15 µg mRNA formulated in 200 µg EV protein per mouse. At 2 h after injection, aortic root sections were collected and stained with DAPI and F4/80. Cy5 signals in aortic root plaques were examined by confocal microscopy to evaluate mRNA delivery to plaque macrophages.

### 5.9 Peripheral Blood Cell Distribution of DiD‐Labeled Vesicles

To evaluate the distribution of DiD‐labeled vesicles among peripheral blood cells, ApoE^−/−^ mice were intravenously injected with PBS, DiD‐labeled Am@Exo, or DiD‐labeled Am@SExo. At 2 h after injection, peripheral blood was collected and processed for flow‐cytometric analysis. Erythrocytes and leukocytes were stained and analyzed in two separate tubes. DiD positivity was quantified in major blood cell populations, including CD11b^+^ myeloid cells, CD3e^+^ T cells, B220^+^ B cells, and Ter119^+^ erythrocytes. The detailed gating strategy is shown in Figure S9, and antibody information is provided in Table S3.

### 5.10 Evaluation of CD47–SIRPα Blockade In Vitro

Jurkat cells were treated with staurosporine (STS) for 2 h to induce apoptosis. The apoptotic cells were then incubated with various EV‐based formulations (final mRNA concentration: 0.5 µg/mL) for 30 min. After incubation, the mixtures were centrifuged to remove unbound materials, and the resulting apoptotic cell–EV complexes (AC–EVs) were collected for subsequent experiments. Fluorescence intensity was measured using flow cytometry (BD FACS Canto II, BD Biosciences, USA), and the mean fluorescence intensity (MFI) of each group was analyzed.

### 5.11 Assessment of Endosomal Escape of mRNA

Efficient cytoplasmic delivery of mRNA requires successful endosomal escape [40]. Bone marrow‐derived macrophages (BMDMs) were seeded in confocal culture dishes (Costar) at a density of 1 × 10^5^ cells per well and cultured for 4 days. Cells were then incubated with Cy5‐labeled mRNA‐loaded exosomes (Exo or SExo) at a final mRNA concentration of 0.5 µg/mL for 1, 4, or 8 h. After incubation, cells were stained with DAPI (Beyotime) to label nuclei and LysoTracker Green (Beyotime) to label acidic endo/lysosomal compartments. Fluorescence signals were analyzed by confocal laser scanning microscopy (CLSM).

### 5.12 Western Blotting Assay

To determine the time‐dependent expression of Arg1, bone marrow‐derived macrophages (BMDMs) were seeded into 6‐well plates at a density of 5 × 10^5^ cells per well. AC–EV complexes were then added at a dose equivalent to 50 µg EV protein per 10^6^ cells. Cells were harvested at different time points, and the expression level of Arg1 protein was analyzed by Western blot to assess its temporal dynamics. To evaluate Arg1 protein expression among different treatment groups, BMDMs (5 × 10^5^ cells/well) were seeded into 6‐well plates and treated with AC–EVs. At the time point corresponding to peak Arg1 expression, the supernatant was discarded, and cells were washed three times with PBS. Cellular proteins were then collected and analyzed by Western blot.

### 5.13 In Vitro Continual Efferocytosis Assay

The procedure for assessing continual efferocytosis was adapted from previously published studies [14, 41]. Apoptotic cells (ACs) were co‐incubated with macrophages at a ratio of 5:1. As shown in Figure 3A, Exo and SExo were respectively loaded with EGFP or Arg1‐EGFP mRNA. The resulting EV–AC mixtures were then added to the culture supernatant of BMDMs or Raw264.7 cells at a dose equivalent to 50 µg EV protein per 10^6^ cells. EGFP^+^ cells were identified as macrophages that had successfully undergone the first round of efferocytosis. After 16 h of incubation, cells were washed three times with PBS and then incubated with DiD‐labeled apoptotic cells (second‐round ACs) for 2 h. Unbound ACs were removed by washing with PBS three times. Cells were then collected for flow cytometry analysis or confocal laser scanning microscopy imaging.

### 5.14 Pro‐Resolving Effect In Vitro

Previous studies have shown that EVs from unpolarized macrophages can also exhibit pro‐resolving activity [42, 43], which motivated our in‐vitro evaluation. BMDMs were subjected to the same treatment protocol as described above. TRIzol was used to extract total RNA, followed by qRT‐PCR to measure transcripts of IL‐10, Arg1, TGF‐β, TNF‐α, IL‐1β, and IL‐6. Primer sequences are listed in Table S1.

### 5.15 In Vivo Treatment and Histological Analysis

ApoE^−^/^−^ mice fed a Western diet (WD) for 4 weeks were intravenously injected twice per week with PBS, Exo, SExo, Am@Exo (15 µg Arg1 mRNA per mouse), or Am@SExo (15 µg Arg1 mRNA per mouse) for a total of 8 weeks. Unloaded EV groups received the same amount of EV protein (200 µg per mouse). For mRNA‐loaded EV treatment groups, each mouse received 15 µg Arg1 mRNA formulated in 200 µg EV protein per injection. This dose was selected based on previously published studies [32, 44]. WD feeding was continued throughout the treatment period. On the third day after the final injection, mice were euthanized and perfused with cold 4% paraformaldehyde (PFA) in PBS. Serial 6 µm‐thick sections of the aortic root were collected for analysis. Oil Red O (ORO, Servicebio) staining was performed to evaluate plaque area, hematoxylin and eosin (H&E) staining was used to assess necrotic core size, and Masson's trichrome staining was used to evaluate fibrous cap thickness. Quantitative analysis was performed using ImageJ software.

### 5.16 In Vivo Efferocytosis Assay

Tissue sections (6 µm) were stained with TUNEL reagent and F4/80. Sections were fixed with 4% PFA and blocked with 5% BSA. F4/80 antibody was incubated overnight, followed by washing and incubation with Alexa Fluor 647 at room temperature for 1 h. TUNEL staining was performed at 37°C for 1 h, followed by DAPI staining. Images were acquired using an Olympus FV3000 confocal microscope, and at least 5 sections per mouse from 5 mice per group were analyzed. For quantitative analysis, TUNEL^+^ nuclei within F4/80^+^ macrophages (appearing purple due to co‐localization of red and blue signals) were identified as apoptotic macrophages. Macrophage‐associated apoptotic cells (ACs) were defined as TUNEL^+^ nuclei that were either surrounded by or in direct contact with F4/80^+^ macrophages. Free apoptotic cells were defined as condensed TUNEL^+^ nuclei not in contact with F4/80^+^ macrophages and lacking F4/80 signal.

Efferocytic capacity was quantified by calculating two parameters. The ratio of TUNEL^+^ nuclei within macrophages to the total number of macrophages (apoptotic MΦ/total MΦ), reflecting macrophage apoptosis. The ratio of macrophage‐associated ACs to free ACs, reflecting in vivo efferocytosis efficiency.

### 5.17 Statistical Analysis

The statistical analysis relied on Student's t‐test, one‐way and two‐way analysis of variance (ANOVA) methods using GraphPad Prism (v10.0). *p* < 0.05 was considered statistically significant. All data were presented as the mean value ± s.d.

## Author Contributions

**Danwen Zheng**: conceptualization, investigation, writing – original draft. **Shiteng Cai**: writing – original draft, investigation, conceptualization. **Jinfeng Gao**: conceptualization, writing – review and editing, investigation. **Sheng Zhao**: methodology, formal analysis. **Bohan Wei**: validation, data curation. **Xueyi Weng**: data curation. **Zhengmin Wang**: visualization. **Qiaozi Wang**: data curation, resources. **Qiyu Li**: visualization, formal analysis. **Chengzhi Han**: visualization. **Weiyan Li**: validation. **Yiwen Tan**: resources. **Yuyuan Fu**: visualization. **Meng Ji**: supervision, project administration. **Zheyong Huang**: writing – review and editing, supervision, funding acquisition. **Yanan Song**: funding acquisition, writing – review and editing, supervision. **Juying Qian**: funding acquisition. Junbo Ge: funding acquisition.

## Funding

This work was supported by the Noncommunicable Chronic Diseases‐National Science and Technology Major Project (2024ZD0537800), the National Natural Science Foundation of China (82370257, 82470263), and the Fujian Joint Fund for Scientific and Technological Innovation Projects (Grant No. 2023J05298).

## Conflicts of Interest

The authors declare no conflicts of interest.

## Supporting information




**Supporting File**: advs75941‐sup‐0001‐SuppMat.docx.

## Data Availability

The data that support the findings of this study are available from the corresponding author upon reasonable request.
