# Supplementary material for: A Dual‐Phase Exosomal Nanotherapy Enhances Continual Efferocytosis by Coordinating Checkpoint Inhibition and Metabolic Reprogramming in Atherosclerosis
Source: Adv Sci (Weinh). 2026 Jun 4;13(48):e75941. doi: 10.1002/advs.75941 (PMC13336402; doi:10.1002/advs.75941)
Supplement: Supplementary file 1 [file ADVS-13-e75941-s001.docx]

**Supporting Information**

**A dual-phase exosomal nanotherapy enhances continual efferocytosis by coordinating checkpoint inhibition and metabolic reprogramming in atherosclerosis**

**Table. S1. Primer sequences of qRT-PCR.**

Gene Primer sequences

GAPDH

Forward: 5’-CCATGGGGAAGGTGAAGGTC-3’

Reverse: 5’-AGTGATGGCATGGACTGTGG-3’

IL-10

Forward: 5’-CGGGAAGACAATAACTGCACCC-3’

Reverse: 5’-CGGTTAGCAGTATGTTGTCCAGC-3’

TNF-α

Forward: 5’-CCACCACGCTCTTCTGTCTA-3’

Reverse: 5’-AGGGTCTGGGCCATAGAACT-3’

IL-1β

Forward: 5’-TGGACCTTCCAGGATGAGGACA-3’

Reverse: 5’-GTTCATCTCGGAGCCTGTAGTG-3’

IL-6

Forward: 5’-CTGCAAGAGACTTCCATCCAG-5’

Reverse: 5’-AGTGGTATAGACAGGTCTGTTGG-3’

**Table S2. Nucleotide sequences of Arg1 and EGFP mRNAs used for qPCR primer design.**

| Arg1 | ATGAGCTCCAAGCCAAAGTCCTTAGAGATTATCGGAGCGCCTTTCTCAAAAGGACAGCCTCGAGGAGGGGTAGAGAAAGGCCCTGCAGCACTGAGGAAAGCTGGTCTGCTGGAAAAACTTAAAGAAACAGAGTATGACGTGAGAGACCACGGGGACCTGGCCTTTGTTGATGTCCCTAATGACAGCTCCTTTCAAATTGTGAAGAACCCACGGTCTGTGGGGAAAGCCAATGAAGAGCTGGCTGGTGTGGTGGCAGAGGTCCAGAAGAATGGAAGAGTCAGTGTGGTGCTGGGTGGAGACCACAGTCTGGCAGTTGGAAGCATCTCTGGCCACGCCAGGGTCCACCCTGACCTATGTGTCATTTGGGTGGATGCTCACACTGACATCAACACTCCCCTGACAACCAGCTCTGGGAATCTGCATGGGCAACCTGTGTCCTTTCTCCTGAAGGAACTGAAAGGAAAGTTCCCAGATGTACCAGGATTCTCCTGGGTGACTCCCTGCATATCTGCCAAAGACATCGTGTACATTGGCTTGCGAGACGTAGACCCTGGGGAACACTATATAATAAAAACTCTGGGAATTAAGTATTTCTCCATGACTGAAGTAGACAAGCTGGGGATTGGCAAGGTGATGGAAGAGACCTTCAGCTACCTGCTGGGAAGGAAGAAAAGGCCGATTCACCTGAGCTTTGATGTCGACGGGCTGGACCCAGCATTCACCCCGGCGACCGGCACCCCGGTTCTGGGAGGCCTATCTTACAGAGAAGGTCTCTACATCACAGAAGAAATTTACAAGACAGGGCTCCTTTCAGGACTAGATATCATGGAAGTGAACCCAACTCTTGGGAAGACAGCAGAGGAGGTGAAGAGTACTGTGAACACGGCAGTGGCTTTAACCTTGGCTTGCTTCGGAACTCAACGGGAGGGTAACCATAAGCCAGGGACTGACTACCTTAAACCACCTAAGTGA |
| --- | --- |
| EGFP | ATGGTGAGCAAGGGCGAGGAGCTGTTCACCGGGGTGGTGCCCATCCTGGTCGAGCTGGACGGCGACGTAAACGGCCACAAGTTCAGCGTGTCCGGCGAGGGCGAGGGCGATGCCACCTACGGCAAGCTGACCCTGAAGTTCATCTGCACCACCGGCAAGCTGCCCGTGCCCTGGCCCACCCTCGTGACCACCCTGACCTACGGCGTGCAGTGCTTCAGCCGCTACCCCGACCACATGAAGCAGCACGACTTCTTCAAGTCCGCCATGCCCGAAGGCTACGTCCAGGAGCGCACCATCTTCTTCAAGGACGACGGCAACTACAAGACCCGCGCCGAGGTGAAGTTCGAGGGCGACACCCTGGTGAACCGCATCGAGCTGAAGGGCATCGACTTCAAGGAGGACGGCAACATCCTGGGGCACAAGCTGGAGTACAACTACAACAGCCACAACGTCTATATCATGGCCGACAAGCAGAAGAACGGCATCAAGGTGAACTTCAAGATCCGCCACAACATCGAGGACGGCAGCGTGCAGCTCGCCGACCACTACCAGCAGAACACCCCCATCGGCGACGGCCCCGTGCTGCTGCCCGACAACCACTACCTGAGCACCCAGTCCGCCCTGAGCAAAGACCCCAACGAGAAGCGCGATCACATGGTCCTGCTGGAGTTCGTGACCGCCGCCGGGATCACTCTCGGCATGGACGAGCTGTACAAGTAA |

**Table S3.** **The detailed list of antibodies used in the study.**

Primary antibodies:

| Antibodies | Company | Host | Lot | Application | Dilution |
| --- | --- | --- | --- | --- | --- |
| SIRP alpha/SHPS1 (D6I3M) Rabbit Monoclonal Antibody | CST | Rabbit | 13379 | WB | 1:1000 |
| CD9 (D8O1A) Rabbit Monoclonal Antibody | CST | Rabbit | 13174 | WB | 1:1000 |
| CD63 (E1W3T) Rabbit Monoclonal Antibody | CST | Rabbit | 52090 | WB | 1:1000 |
| TSG101 (E6V1X) Rabbit Monoclonal Antibody | CST | Rabbit | 72312 | WB | 1:1000 |
| Calnexin (C5C9) Rabbit Monoclonal Antibody | CST | Rabbit | 2679 | WB | 1:1000 |
| Arginase-1 (D4E3M) Rabbit Monoclonal Antibody | CST | Rabbit | 93668 | WB | 1:1000 |
| GAPDH (14C10) Rabbit Monoclonal Antibody (HRP Conjugate) | CST | Rabbit | 3683 | WB | 1:5000 |
| CD18 Recombinant monoclonal antibody | Proteintech | Rabbit | 87481-2-RR | WB | 1:1000 |
| Anti-CCR2 antibody | Abcam | Rabbit | Ab273050 | WB | 1:1000 |
| CXCR2 Recombinant monoclonal antibody | Proteintech | Rabbit | 85144-5-RR | WB | 1:1000 |
| F4/80 (D4C8V) Rabbit Monoclonal Antibody | CST | Rabbit | 30325 | IF | 1:200 |

Fluorophore conjugated primary antibodies:

| Antibodies | Company | Host | Lot | Application | Dilution |
| --- | --- | --- | --- | --- | --- |
| APC-Cy7-anti-CD45 | Biolegend | Rat | 103116 | Flow cytometry | 1:100 |
| FITC-anti-CD45 | Biolegend | Rat | 147710 | Flow cytometry | 1:100 |
| PerCP-Cy5.5-anti-CD11b | Biolegend | Rat | 101228 | Flow cytometry | 1:100 |
| PE-anti-F4/80 | Biolegend | Rat | 123145 | Flow cytometry | 1:100 |
| PE-Cy7-anti-CD86 | BD pharmingen | Rat | 560582 | Flow cytometry | 1:100 |
| APC-anti-CD206 | Invitrogen | Rat | 3135958 | Flow cytometry | 1:100 |
| FITC-anti-CD47 | Biolegend | Rat | 127503 | Flow cytometry | 1:100 |
| FITC-anti-TER-119 | Biolegend | Rat | 116205 | Flow cytometry | 1:100 |
| PE-anti-CD45R/B220 | Biolegend | Rat | 103207 | Flow cytometry | 1:100 |
| FITC-anti-CD3e | Biolegend | Rat | 100305 | Flow cytometry | 1:100 |

Secondary antibodies:

| Antibodies | Company | Host | Lot | Application | Dilution |
| --- | --- | --- | --- | --- | --- |
| Alexa Fluor 488 goat anti-rabbit IgG | Thermo Fisher | Goat | A-11008 | IF | 1:500 |
| Alexa Fluor 568 goat anti-rabbit IgG | Thermo Fisher | Goat | A-11011 | IF | 1:500 |
| HRP-conjugated goat anti-rabbit IgG | CST | Goat | 7074 | WB | 1:5000 |

**Table S4. Detailed list of dyes and staining reagents used in this study.**

| Reagent | Company | Lot | Dilution |
| --- | --- | --- | --- |
| DiO | Servicebio | G1704 | 1:250 |
| DiD | Invitrogen | D7757 | 1:1000 |
| TUNEL staining kit | Beyotime | C1090 | According to manufacturer’s instructions |
| LysoTracker Green | Beyotime | C1047S | 1:10000 ~ 1:20000 |
| WGA, Texas Red conjugate | Invitrogen | W21405 | 1:200 |
| ICAM antibody | Santa Cruz Biotechnology | sc-8439 | 1:200 |
| Phalloidin | ABclonal | RM02836 | 1:200 |

**Figure S1.** Western blot analysis of SIRPα expression in Raw264.7 untreated or transduced with empty vector, or SIRPα-overexpressing lentivirus at MOI = 200 and 400. Data are presented as mean ± s.d. (n = 3). Statistical significance was determined using a two-sided Student’s t-test.

**Figure S2.** Confocal microscopy images of Raw264.7 cells transduced with control lentivirus (V0) or SIRPα-expressing lentivirus at MOI = 200 (S200), showing EGFP fluorescence (green) and nuclear staining with DAPI (blue). Scale bar, 50μm.

**Figure S3.** Purified IVT mRNA production was confirmed by electrophoresis on an agarose gel.

**Figure S4. Encapsulation of Arg1 mRNA within EVs.**

The mRNA encapsulation efficiency of different EVs was calculated as: **(total input mRNA − unencapsulated mRNA in the supernatant) / total input mRNA concentration.** Data are presented as mean ± SD (n=3). Statistical significance was determined using a two-sided Student’s t-test.

**Figure S5. Stability evaluation of Am@SExo and its protection of encapsulated mRNA against serum and RNase degradation.**

(A) Particle size of Am@SExo after incubation in 10% serum for the indicated times. (B) Polydispersity index (PDI) of SExo and Am@SExo. Data are presented as mean ± SD (n=3). Statistical significance was determined using a two-sided Student’s t-test.

N.S., not significant.

(C) Agarose gel electrophoresis analysis of naked Arg1 mRNA and Am@SExo after exposure to 10% serum or RNase A for the indicated times.

**Figure S6. Cellular uptake of engineered EVs by RAW264.7 and BMDMs.**

(A) Representative CLSM images of Raw264.7 cells incubated with DiD-labeled Exo or SExo for 30min.

(B) Representative CLSM images of bone marrow-derived macrophages (BMDMs) treated under the same conditions. Cell nuclei were stained with DAPI (blue), and EVs were labeled with DiD (red). Scale bar, 25 μm.

**Figure S7. Characterization of surface adhesion molecules on Exo and SExo.**

Western blot analysis of CD18, CCR2, and CXCR2 on Exo and SExo, with CD9 used as an exosomal marker for normalization. Quantitative analysis of protein expression levels (CXCR2/CD9, CD18/CD9, and CCR2/CD9) between Exo and SExo groups (n = 3). Data are presented as mean ± SD. Statistical significance was determined using a two-sided Student’s t-test.

**Figure S8. Biodistribution and plaque-targeting ability of SExo and Am@SExo.**

(A) Ex vivo IVIS images of major organs harvested 2 h after intravenous injection of PBS or DiD-labeled EVs into 12-week Western diet (WD)-fed ApoE^-/-^ mice.

(B) Quantification analysis of fluorescence signal in aortas and major organs. Statistical analysis was calculated using the one-way ANOVA and Tukey’s multiple comparison tests. (n = 4).

(C) Aorta-to-liver fluorescence ratio. Statistical analysis was calculated using the one-way ANOVA and Tukey’s tests (n = 4).

(D) Immunofluorescence analysis of Cy5-labeled mRNA delivery in the aortic root after treatment with PBS, Am@Exo, or Am@SExo. Cy5-labeled mRNA is shown in red, F4/80⁺ macrophages in green, and nuclei in blue. White arrows indicate Cy5 signals associated with F4/80⁺ macrophages. Scale bar, 25 μm.

**Figure S9. Flow-cytometric analysis of DiD-labeled EV distribution in circulating blood cells 2 h after intravenous injection.**

(A) Gating strategy for leukocyte populations. Live cells were first gated, followed by CD45^+^ leukocytes, and then subdivided into CD11b^+^ myeloid cells, CD3e^+^ T cells, and B220^+^ B cells. DiD positivity was subsequently quantified in each population.

(B) Gating strategy for erythrocytes. Live cells were gated and Ter119^+^ erythrocytes were identified, followed by quantification of DiD^+^ events.

(C) Representative flow-cytometry plots showing DiD signal in CD11b^+^ myeloid cells, CD3e^+^ T cells, B220^+^ B cells, and Ter119^+^ erythrocytes from mice treated with PBS, Am@Exo, or Am@SExo.

(D) Quantification of DiD^+^ percentages in CD11b^+^ myeloid cells, CD3e^+^ T cells, B220^+^ B cells, and Ter119^+^ erythrocytes. Data are presented as mean ± SD. Statistical analysis was calculated using the one-way ANOVA and Tukey’s tests (n = 4). N.S., not significant.

**Figure S10. Flow-cytometric analysis of macrophage uptake of DiD-labeled EVs.**

(A) Gating strategy.

(B) Representative flow-cytometry plots showing DiD signals in macrophages after treatment with PBS, Exo, SExo, Am@Exo, or Am@SExo.

(C) Quantification of DiD⁺ macrophages among total macrophages. Statistical analysis was calculated using the one-way ANOVA and Tukey’s tests(n = 3). N.S., not significant.

**Figure S11.** **Representative CLSM images of BMDM undergoing two rounds of efferocytosis.** Scale bar, 10 μm.

**Figure S12. Flow cytometry analysis of two-round efferocytosis in Raw264.7 and BMDMs.**

(A) Representative flow cytometry plots showing single (EGFP⁺) and continual (EGFP⁺DiD⁺) efferocytosis in Raw264.7 cells treated with Exo, SExo, Am@Exo, or Am@SExo.

(B) Representative flow cytometry plots showing the sequential gating strategy for BMDMs after two rounds of efferocytosis. Red arrows indicate the gating order from live cells to CD45⁺ cells, CD11b⁺F4/80⁺ macrophages, and finally EGFP- or DiD-positive populations.

(C–D) Quantification of single and continual efferocytosis in BMDMs based on EGFP and DiD fluorescence. Statistical analysis was calculated using the one-way ANOVA and Tukey’s tests (n = 3).

**Figure S13. Quantitative PCR analysis of pro-inflammatory and pro-resolving cytokine expression in BMDMs after efferocytosis.**

BMDMs were treated with PBS, Exo, SExo, Am@Exo, or Am@SExo and subjected to efferocytosis. The relative mRNA expression levels of pro-resolving markers (Arg1, TGF-β, IL-10) and pro-inflammatory cytokines (TNF-α, IL-1β, IL-6) were measured by qPCR. Data are presented as mean ± SD. Statistical analysis was calculated using the one-way ANOVA and Tukey’s tests (n = 3).

**Figure S14. Representative flow-cytometry plots corresponding to Figures 5B and 5C.**

BMDMs were treated with PBS, Exo, SExo, Am@Exo, or Am@SExo, followed by two rounds of efferocytosis. Representative plots show the sequential gating strategy used for macrophage polarization analysis, including live cells, CD45⁺ cells, CD11b⁺F4/80⁺ macrophages, and subsequent assessment of CD86 and CD206 expression. Red arrows indicate the gating sequence.

**Figure S15. Targeted metabolomic analysis of key metabolites in the arginine metabolism pathway after Am@SExo treatment.**

Relative abundances of targeted metabolic substances in BMDMs were measured by targeted metabolomics following treatment with PBS or Am@SExo. Data are shown as box plots representing relative metabolite abundance. Statistical significance was determined using unpaired two-sided Student’s *t*-test (n = 4).

**Figure S16. qPCR analysis of key enzymes involved in arginine metabolism in BMDMs after efferocytosis.**

BMDMs were treated with PBS, Exo, SExo, Am@Exo, or Am@SExo and subjected to efferocytosis. The mRNA expression levels of key enzymes related to arginine metabolism, including ornithine decarboxylase (ODC), Rac1, were measured by quantitative PCR. Data are presented as mean ± SD. Statistical analysis was performed using one-way ANOVA and Tukey’s tests (n = 3).

**Figure.S17.** Complete Oil Red O staining images corresponding to Figures 6B and 6D.

**Figure S18. Histological evaluation of systemic toxicity after 4-week Am@SExo treatment in ApoE^⁻/⁻^ mice.**

ApoE^⁻/⁻^ mice were treated with PBS or Am@SExo via tail vein injection once every 3 days for 4 weeks. Three days after the final injection, mice were euthanized, and major organs—including the heart, liver, spleen, lungs, kidneys, and brain—were harvested and subjected to hematoxylin and eosin (H&E) staining. Scale bar, 20 μm.
